# Flavonoid Biosynthesis Is Likely More Susceptible to Elevation and Tree Age Than Other Branch Pathways Involved in Phenylpropanoid Biosynthesis in *Ginkgo* Leaves

**DOI:** 10.3389/fpls.2019.00983

**Published:** 2019-07-30

**Authors:** Kai Zou, Xueduan Liu, Du Zhang, Qin Yang, Shaodong Fu, Delong Meng, Wenqi Chang, Rui Li, Huaqun Yin, Yili Liang

**Affiliations:** ^1^School of Minerals Processing and Bioengineering, Central South University, Changsha, China; ^2^Key Laboratory of Biometallurgy of Ministry of Education, Changsha, China; ^3^State Key Laboratory of Natural Medicines, Department of Chinese Medicines Analysis, China Pharmaceutical University, Nanjing, China; ^4^Zhejiang CONBA Pharmaceutical, Co., Ltd., Hangzhou, China

**Keywords:** *Ginkgo biloba*, flavonoid, non-flavonoids polyphenolics, transcriptome, LC–MS, elevation, plant age

## Abstract

*Ginkgo* leaves are always resources for flavonoids pharmaceutical industry. However, the effect of the elevation and tree age changes on flavonoid biosynthesis have not been detailly explored in *Ginkgo* leaves. In addition, whether these environmental pressures have similar effects on the biosynthesis of other non-flavonoids polyphenolics in phenylpropanoid biosynthesis is not known at present. In this research, *de novo* transcriptome sequencing of *Ginkgo* leaves was performed coupled with ultra-performance liquid chromatography/quadrupole time-of-flight mass spectrometry analyses to obtain a comprehensive understanding of the influence of elevation and tree age on phenylpropanoid biosynthesis. A total of 557,659,530 clean reads were assembled into 188,155 unigenes, of which 135,102 (71.80%) were successfully annotated in seven public databases. The putative *DFR*s, *LAR*s, and *ANR*s were significantly up-regulated with the increase of elevation in young *Ginkgo* tree leaves. The relative concentration of flavonoid derivatives with high parent ion intensity was likely to imply that the elevation increase promoted the biosynthesis of flavonoids. Complex gene variations involved in flavonoid biosynthesis were observed with the tree age increase. However, flavonoid derivatives analysis predicted that the rise of tree age was more likely to be detrimental to the flavonoids manufacture. Otherwise, multiple genes implicated in the synthesis of hydroxycinnamates, lignin, and lignan exhibited fluctuations with the elevation increase. Significantly up-regulated *CAD*s and down-regulated *PRD*s potentially led to the accumulation of *p*-Coumaryl alcohol, one of the lignin monomers, and might inhibit further lignification. Overall, the putative *DFR*s seemed to show more considerable variability toward these stress, and appeared to be the main regulatory point in the flavonoid biosynthesis. Light enhancement caused by elevation increase may be the main reason for flavonoids accumulation. Flavonoid biosynthesis exhibited a greater degree of perturbation than that of hydroxycinnamates, lignins and lignans, potentially suggesting that flavonoid biosynthesis might be more susceptible than other branch pathways involved in phenylpropanoid biosynthesis. This research effectively expanded the functional genomic library and provide new insights into phenylpropanoid biosynthesis in *Ginkgo*.

## Introduction

Phenylpropanoids biosynthesis pathway functions as a natural factory to produce a variety of secondary metabolites in response to biological and abiotic stimuli (Deng and Lu, 2017). Derived from phenylalanine in most plants or tyrosine in partial monocots, the central phenylpropanoids mainly include flavonoids, monolignols, hydroxycinnamates (HCAs), lignins, and lignans, which acting as components of cell walls, protectants against UV radiation, signaling molecules phytoalexins against herbivores and pathogens (Vogt, 2010; Deng and Lu, 2017).

Generally, the first enzymatic step of phenylpropanoid biosynthesis begins with the deamination of phenylalanine by phenylalanine ammonia lyase (PAL) to yield cinnamic acid (Barros et al., 2016). In the second step, cinnamate 4-hydroxylase (C4H) hydroxylates and transforms the cinnamic acid into *p*-coumaric acid. Then, 4-coumaroyl CoA ligase (4CL) catalyzes *p*-coumaric acid into *p*-coumaroyl-CoA, which is a crucial branch point leading to the generation of flavonoids, lignins and lignans (Vogt, 2010).

Plants have evolved a variety of antioxidant systems to cope with reactive oxygen species (ROS) generated under ambient pressures (Brunetti et al., 2015). As an important antioxidant system, phenylpropanoid biosynthesis is regulated by many factors, including light, irrigation, temperature, and fertilization (Kim et al., 2015). Of which, light appears to be the most important factor that affects the dynamic synthesis of multiple polyphenolic substances. For instance, the light of different wavelengths has been widely used to accumulate phenolic compounds in lettuce (Johkan et al., 2010), chrysanthemum (Jeong et al., 2012), and buckwheat (Thwe et al., 2014). Besides, it has been widely reported that the enhancement of light can significantly increase the flavonoids content in fruits (Awad et al., 2001; Azuma et al., 2012; Zoratti et al., 2014). Studies of circadian rhythms show that light signals promote the synthesis of lignin in the night in *Arabidopsis* (Rogers et al., 2005). Lignans were observed a notable increase under continuous white fluorescent and monochromatic red or blue light emitting diode (LED) light (Hata et al., 2013). However, the light-induced fluctuation of HCA biosynthesis shows uneven regularity (Kolb et al., 2001; Hemm et al., 2004).

*Ginkgo biloba* (*Ginkgo*) is widely used as traditional Chinese medicine for centuries, especially on asthma and cardiovascular disease (Kleijnen and Knipschild, 1992; Birks and Grimley Evans, 2007). The flavonoids and terpene trilactones are currently considered the most prominent pharmacological components of *Ginkgo* (van Beek, 2002). The studies on the phenylpropanoid biosynthesis of *Ginkgo* mainly focused on flavonoids, especially the influences of populations (Rimkiene et al., 2017), seasonality (Sati et al., 2013), and growth stages (Mikašauskaitė et al., 2013).

In general, the elevation increase always contributes to light enhancement and a decrease in the average temperature. Little evidence indicated the full impact of variable elevation and tree age on the flavonoid biosynthesis of *Ginkgo* and its possible reasons. The non-flavonoids polyphenolics need further discussion as well. Moreover, few discussions have focused on the effect of tree age on the phenylpropanoids biosynthesis in woody plant leaves. And whether different branches of phenylpropanoid synthesis will show consistent responses to the same environmental stress need more discussion. Therefore, in this study, wild *Ginkgo* trees were sampled to investigate the specific profile changes of phenylpropanoids biosynthesis induced by the variation of elevation and tree age via non-referenced transcriptome analysis coupled with ultra-performance liquid chromatography/quadrupole time-of-flight mass spectrometry.

## Materials and Methods

### Ginkgo Leaves

**FIGURE 1:**
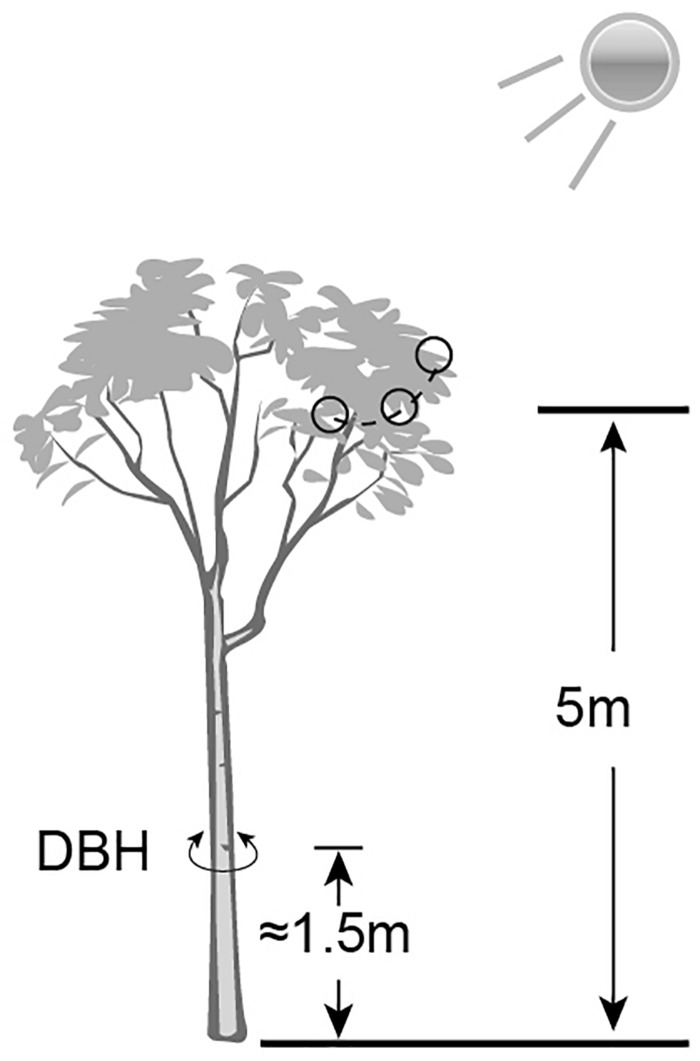
A schematic diagram of sample collection and data measurement. Small black circles indicated the sampling sites.

The *Ginkgo* trees grow wildly in Pot bottles of mountain Nature Reserve in Hunan Province, China. A total of 12 trees (26^∘^55′13′′ N to 30^∘^6′44′′ N, 110^∘^36′16′′ E to 110^∘^49′2′′ E) were chosen to collect mature leaf samples (Supplementary Table S1). According to the formula (age of tree = diameter ^*^ growth factor) proposed by the International Society of Arboriculture in the United States for estimating the age of trees, it can be inferred that the diameter at breast height (DBH) positively correlates with the age of the trees in the same (or similar) environment. So, the DBH (converted from trunk circumference) was used as a measure of the relative extent of tree ages. The elevation and the DBH were determined by Global Positioning System (GPS) instrument and tape measure respectively. All samples were divided into four groups – low elevation and young age (LY), high elevation and young age (HY), low elevation and older age (LO), high elevation and older age (HO). Each group involved three tree individuals. Each sample was mixed by thirty leaves of similarly size and health, which were cut off from the sunny side 5 m above the ground of the same tree (Figure 1). After harvesting and short-time surface cleaning by 75% ethanol and sterile water, these leaves were frozen in liquid nitrogen immediately and stored at −80^∘^C until used. The same copies of each leaf sample were separately used for RNA extracting and UPLC-MS.

### RNA Extraction, Library Preparation, and Sequencing

Total RNA extraction was carried out by E.Z.N.A.^®^ Plant RNA Kit (Omega Bio-Tek, Inc.) according to the manufacturer’s instruction coupled with genomic DNA digesting with DNase I. Then 1% agarose gel electrophoresis was used to monitor RNA degradation and contamination. RNA purity, concentration and inte- grity were measured by NanoPhotometer^®^ spectrophotometer (Implen, Westlake Village, CA, United States), Qubit 2.0 Fluorometer (Life Technologies, Foster City, CA, United States) and Agilent Bioanalyzer 2100 system (Agilent Technologies, Santa Clara, CA, United States). Dynabeads Oligo (dT) (Invitrogen, United States) was used to isolate mRNA from total RNA. Then the NEB Next^®^ Ultra RNA Library Prep Kit for Illumina^®^ (NEB, United States) was used to construct 12 cDNA libraries following manufacturer’s recommendations. After some necessary processes (index-codes adding, mRNA purification, fragmentation, cDNA synthesis, exonuclease/polymerase cleavage, adenylation of 3′ ends of cDNA fragments, adaptor ligation, size selection, PCR and purification) and the terminal quality assessment on the Agilent Bioanalyzer 2100 system, the library preparations were sequenced on an Illumina Hiseq PE150 platform and paired-end reads were generated.

### Quality Control, *de novo* Assembly, and Annotation

Raw data in fastq format (Cock et al., 2010) were processed through in-house Perl scripts. Clean data were obtained by removing reads containing adapter, reads containing ploy-N and low-quality reads from raw data. At the same time, Q20, Q30, GC-content and sequence duplication level of the clean data were calculated. Transcriptome assembly was accomplished by Trinity (v2.4.0) (Grabherr et al., 2011; Haas et al., 2013) with min_kmer_cov set to 1 and all other parameters set default. All transcripts were clustered into unigenes in Corset (v1.05) with all parameters default as recommended (Davidson and Oshlack, 2014). Then, these unigenes were searched against seven databases for function annotation. NCBI blast 2.2.28+ (Altschul et al., 1997) was used for the alignments of unigenes to Nt database with an E-value threshold of 1E-5. The program diamond (v0.8.22) (Buchfink et al., 2015) was selected to perform the comparison against Nr (E-value 1E-5), KOG/COG (E-value 1E-3), and Swiss-Prot (E-value 1E-5) databases. The hmmscan in HMMER 3.0 was operated to search Pfam (Finn et al., 2008) with an E-value threshold of 0.01. The GO (Gene Ontology) annotations were carried out in Blast2GO (v2.5) (Gotz et al., 2008) with an E-value threshold of 1E-6 based on the Nr and Pfam annotations. KAAS^1^ (Moriya et al., 2007) was used for the KEGG annotations (E-value 1E-6). Transcription factors (TFs) was predicted in iTAK 1.2 with all parameters set default (Perez-Rodriguez et al., 2010; Jin et al., 2014; Zheng et al., 2016).

The raw data have been submitted to NCBI SRA database. The accession numbers of all samples were SRR8464455, SRR8464456, SRR8464453, SRR8464454, SRR8464451, SRR8464452, SRR8464449, SRR8464450, SRR8464457, SRR8464458, SRR8464447, and SRR8464448 in Bioproject PRJNA515544. The Trinity assembly has been deposited at DDBJ/EMBL/GenBank under the accession GHLL00000000.

### Gene Expression Levels and Differentially Expressed Genes Identification

The assembled transcriptome spliced by Trinity was set as the reference sequence (ref) (Haas et al., 2013). The clean reads of each sample were mapped back to this ref in RSEM (v1.2.15) (Li and Dewey, 2011) with the Bowtie2 mismatch set 0 as default. The mapping results including read counts of each sample were normalized by calculating the FPKM (expected number of Fragments Per Kilobase of transcript sequence per Millions of base pairs sequenced) to obtain relative expression levels of unigenes (Trapnell et al., 2010) (Supplementary Datasheet S1). An FPKM > 0.3 was defined as the threshold of significant gene expression (Ramskold et al., 2009). Differentially expressed genes (DEGs) identification between different groups was performed by DESeq2 R package (v1.6.3) (Love et al., 2014) after FPKM tables were normalized as recommended (Dillies et al., 2013). The unigenes with an adjusted *p*-value < 0.05 were assigned as DEGs. To figure out the major functional groups of DEGs, GOseq R package was used to implement Gene Ontology (GO) enrichment analysis of the DEGs based on the Wallenius non-central hyper-geometric distribution (Young et al., 2010). KOBAS (Mao et al., 2005) was used to test the statistical enrichment of DEGs in KEGG pathways.

### Quantitative Real-Time PCR Validation

Total RNA was reverse transcribed into cDNA after residual genomic DNA degradation by ReverTra Ace^®^ qPCR RTMaster Mix with gDNA remover Kit (TOYOBO, Japan). Then quantitative PCR experiments were performed on Bio-Rad iQ5 Optical System (BIO-RAD Laboratories, Inc., United States) with KOD SYBR^®^ qPCR Mix Kit (TOYOBO, Japan). Primers used in this process were designed in Primer Premier 6 (United States), and the primer sequences are given in Supplementary Table S2. The relative expression levels were calculated according to the absolute quantification of mRNA based on standard curve line as commonly recommended (Bustin, 2000). Glyceraldehyde 3-phosphate dehydrogenase (GAPDH) gene was used as an internal standard (Meng et al., 2016; Meng and Fricke, 2017). As designed, each group included three biological replicates and each cDNA sample was carried out with three independent technical repetitions.

### LC–MS Samples Preparation

To minimize the error, andrographolide was dissolved in acetonitrile as an internal standard solution with the terminal concentration of 133 μM. Of each leaf sample, 200 mg liquid nitrogen-grinded powder was homogenized in 30 ml 70% ethanol solution (v: v = 70: 30) followed by 1 min vortex and 1 h ultrasound extraction as previously described with minor modifications (Yu et al., 2003; Tohge et al., 2005; Zhou et al., 2014). After a 13000 rpm centrifugation for 10 min at 4^∘^C, 1 ml supernatant of the solution was transferred and evaporated to dryness under nitrogen gas at 37^∘^C (Li et al., 2017). The residue was re-dissolved in 1 ml acetonitrile and centrifuged at 13000 rpm for 10 min at 4^∘^C. 950 μL supernatant was transferred to mix with 50 μL internal standard solution to be the final sample for further UPLC-QTOF/MS analysis.

### UPLC-MS Conditions

Chromatographic separation was completed on a Shimadzu LC-30AD Series UPLC system (Shimadzu, Duisburg, Germany) equipped with supplementary SIL-30AC injector and (PAD) SPD-M20A detector. ACQUITY UPLC^TM^ BEH C18 column (100 mm × 2.1 mm, 1.7 μm, Waters, Milford, MA, United States) was attached to the whole analyses at 35^∘^C. A flow rate of 0.3 mL/min was chosen to use while 0.1% formic acid water (A) and acetonitrile (B) comprising the mobile phase. The gradient elution conditions were optimized as follows: 0–3 min, 5% B → 5% B; 3–25 min, 5% B → 95% B; 25–28 min, 95% B → 95% B; 28–28.1 min, 95% → 5% B, followed by 4 min re-equilibration.

AB SCIEX TripleTOF^TM^ 5600+ system (AB SCIEX Technologies, United States), equipped with an electrospray ionization (ESI) source, was coupled to the UPLC system and used to scan parent ion molecular weight from 100 to 1500. Other MS parameters were set as below: electrospray ionization temperature (^∘^C): 500 (ESI^–^); nebulizer gas pressure (psi): 60 (ESI^–^); ion spray voltage (KV): 4.5 (ESI^–^); collision energies (V): 35 (ESI^–^).

### Quality Control

To monitor the stability and quality of the non-targeted bioanalysis, the quality control (QC) samples were prepared by pooling 0.2 g powder of each sample and extracted in the same method (Chang et al., 2017). Linearity was assessed by calculating coefficients (R^2^) of the diluting of QC samples in series (1,2,5,8,10,20,50,100,200, and 500 times). Repeatability was evaluated by six replicates of QC samples prepared and extracted parallelly as above. Since all tests are completed in 1 day, precision was investigated by intra-day variability, which was inspected by injecting the QC sample six times along with experimental samples detection in 1 day.

### UPLC-MS Data Preprocessing and Biomarkers Identification

The raw data outputted from LC–MS was pretreated by MarkerView (version 1.2.1.1, AB SCIEX Technologies, United States), including peak recognition (retention time 2–28 min, noise threshold 100), alignment, calibration of the internal standard, filtering and normalization to total area. A three-dimensional data set contained sample information, peak retention time (RT), peak relative intensities and mass-to-charge ratio (m/z) was obtained to perform a series of statistical analysis. PeakView (version 1.2.0.3, AB SCIEX Technologies, United States) was recommended to visualize raw data of target components in two-stage mass-to-charge ratio map. Then, based on fragment ion information, such components were identified by comparing to HMDB^2^, PubChem^3^, NIST^4^, MassBank^5^, and METLIN^6^ databases (Linstrom and Mallard, 2001; Horai et al., 2010; Domingo-Almenara et al., 2018; Kim et al., 2018; Wishart et al., 2018).

## Results

### Sequencing and *de novo* Assembly

**FIGURE 2:**
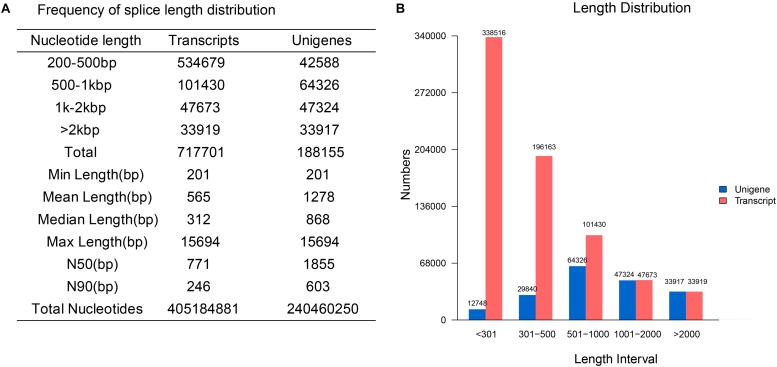
The detailed statistics and distribution of transcripts and unigenes. **(A)** The statistics of transcripts and unigenes. **(B)** The length distribution of transcripts and unigenes.

83.64 Gb high-quality sequences were obtained from sequencing and pretreatment (Supplementary Table S3) with 0.01–0.02% error rates. The average Q20 and Q30 were 96.94 and 92.37%. The average GC content was 46.51%. All the clean reads were pooled together for *de novo* assembly in Trinity (Grabherr et al., 2011). A total of 188,155 unigenes with a mean length of 1,278 bp and an N50 of 1,855 bp were generated, among which 81,241 unigenes (43.18%) were longer than 1,000 bp. The detailed length distribution of the transcripts and unigenes is shown in Figure 2.

### Functional Annotation and Classification

There were 135,102 unigenes, accounting for 71.8%, annotated in at least one database, and 17,500 (9.3%) unigenes were observed matches in all seven databases (Supplementary Table S4 and Supplementary Datasheet S2). The Nr database had the highest percentage (60.64%) of similar entries.

GO classification indicated that numerous terms were assigned under 25 subcategories of biological process, 21 subcategories of cellular component, and 10 subcategories of molecular function in GO classification (Supplementary Figure S1a). For KOG classification (Supplementary Figure S1b), all annotated unigenes were most clustered in (O) Post-translational modification, protein turnover, chaperones, followed by (J) Translation, ribosomal structure and biogenesis, and (R) General function prediction only. The KEGG pathway database was always used to predict the interactions of the genes and the products. Overall, 51,137 unigenes were functionally classified into 132 pathways corresponding to multiple secondary hierarchies (Supplementary Figure S1c).

### Gene Expression Statistics and DEGs Analysis

All clean reads of each sample were analyzed back to the transcriptome assembly. As listed in Supplementary Table S5, over 70% of the sequences per sample were matched, which indicated reliable data processing and quality. Density distributions of gene expressions in each group were shown in Supplementary Figure S2, where the difference between LO and HO was the most significant. Significant transcriptional differences between all groups were reflected in the PCA plot (Supplementary Figure S3).

HY vs. LY and HO vs. LO were used to predict the effect of elevation on flavonoid biosynthesis in *Ginkgo* leaves at two plant age levels. Parallelly, LO vs. LY and HO vs. HY were selected to evaluate the effect of plant age on flavonoid biosynthesis in *Ginkgo* leaves at two elevation levels.

Volcano plots in Supplementary Figure S4 revealed that 1,264 DEGs were observed when the elevation raised in HY vs. LY, including 547 up-regulated and 717 down-regulated unigenes (Supplementary Table S6). And in HO vs. LO, 2,046 DEGs were observed, 186 up-regulated and 1,860 down-regulated. Venn diagrams (Supplementary Figure S5) showed that 46 DEGs were identified in both HY vs. LY and HO vs. LO, of which 10 DEGs were up-regulated and 34 DEGs were down-regulated in both comparisons. In terms of quantity, it seemed that the high age of *Ginkgo* trees significantly affected the response of genes expression to elevation change, mainly reflected in the significant increase of down-regulated unigenes.

As to the influence of plant age, 6,438 DEGs were filtered out from LO vs. LY, 4,915 of which were up-regulated and 1,523 of which were down-regulated. However, only a total of 139 DEGs were found in HO vs. HY, including 79 DEGs up-regulated and 60 DEGs down-regulated. Thereby, there were only nine DEGs observed both in LO vs. LY and HO vs. HY with two up-regulated and two down-regulated synchronously. Obviously, the higher elevation appeared to make gene expression insensitive to the change of *Ginkgo* trees age.

### GO Enrichment of DEGs

Thirty most enriched GO categories of each comparison were plotted in Supplementary Figure S6. The results showed that the DEGs in HY vs. LY were significantly enriched in “obsolete peroxidase reaction” (GO:0006804) of biological process, “peroxidase activity” (GO:0004601) and “oxidoreductase activity, acting on peroxide as acceptor” (GO:0016684) of molecular function. The independent enrichment of up-regulated unigenes indicated a significant enhancement of hormone regulation and metabolism due to the rise of elevation in HY vs. LY, especially in “regulation of hormone levels” (GO:0010817), “cellular hormone metabolic process” (GO:0034754) and “hormone metabolic process” (GO:0042445), which was hypothesized to play an essential role in the regulation of flavonoid biosynthesis (Brunetti et al., 2018). However, in HO vs. LO, there were 65 enriched categories, of which “catalytic activity” (GO:0003824) was enriched the most in molecular function, and “carboxylic acid metabolic process” (GO:0019752) was enriched the most in biological process, while “tricarboxylic acid cycle enzyme complex” (GO:0045239) was the only enriched category in cellular component. The down-regulated unigenes were significantly enriched in decentralized GO terms, including multiple terms relate to the biological process of nucleic acid. It seemed that the older *Ginkgo* trees need more nucleic acids process to cope with the abiotic stress caused by elevation change.

In LO vs. LY, 45 categories in biological process, 10 categories in cellular component and 49 categories in molecular function were observed significant enrichment. “Catalytic activity” (GO:0003824) was the most enriched category in molecular function, followed by “pyrophosphatase activity” (GO:0016462) and “hydrolase activity, acting on acid anhydrides, in phosphorus-containing anhydrides” (GO:0016818). “Localization” (GO:0051179) was the most enriched category in biological process, followed by “transport” (GO:0006810) and “establishment of localization” (GO:0051234). “Holliday junction helicase complex” (GO:0009379) was the most enriched category in cellular component, followed by “Holliday junction resolvase complex” (GO:0048476) and “DNA helicase complex” (GO:0033202). Further, the up-regulated unigenes were enriched in 120 categories while the down-regulated ones enriched in nine categories. Nevertheless, no categories were significant enriched in HO vs. HY, although “toxic substance binding” (GO:0015643) of molecular function and “immunoglobulin complex” (GO:0019814) of cellular component possessed the lowest corrected *p*-values. Moreover, “binding” (GO:0005488) was the most abundant category with 78 DEGs, followed by “cellular process” (GO:0009987) and “metabolic process” (GO:0008152).

### KEGG Enrichment of DEGs

All DEGs, as well as the up- and down-regulated DEGs separately, were mapped to the KEGG database to identify genes involved in signal transduction or metabolic pathways. Twenty pathways with the most enrichment degree of each comparison were shown in bar plots.

**FIGURE 3:**
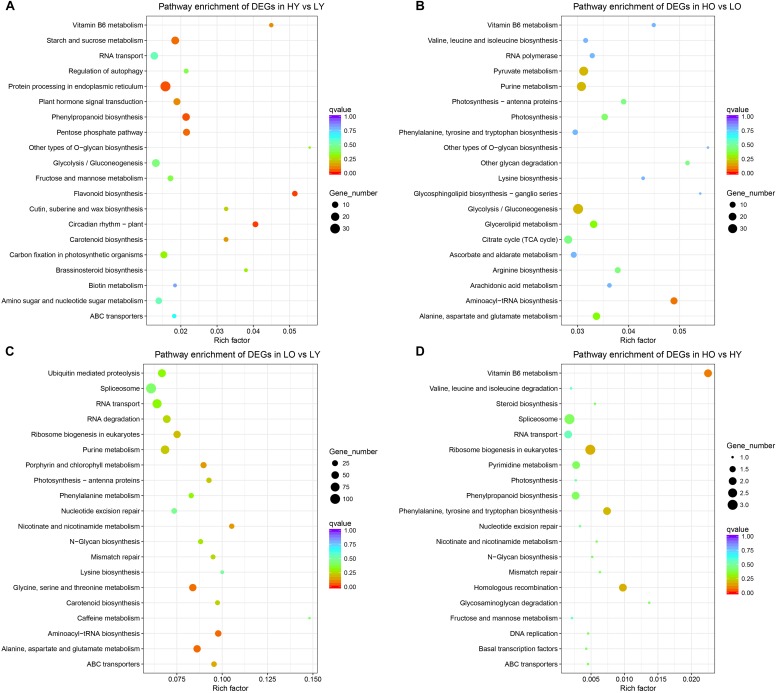
KEGG pathway enrichment of all comparisons. **(A)** The most enriched KEGG pathways of DEGs in HY vs. LY. **(B)** The most enriched KEGG pathways of DEGs in HO vs. LO. **(C)** The most enriched KEGG pathways of DEGs in LO vs. LY. **(D)** The most enriched KEGG pathways of DEGs in HO vs. HY. The rich factor stands for the ratio of the DEGs number and the number of genes annotated in this pathway. The larger of the rich factor means the greater degree of enrichment.

All DEGs of HY vs. LY (Figure 3A) were most enriched in “flavonoid biosynthesis” (ko00941), followed by “circadian rhythm – plant” (ko04712), “phenylpropanoid biosynthesis” (ko00940) and “protein processing in endoplasmic reticulum” (ko04141). “O-glycan biosynthesis” (ko00514) and “flavonoid biosynthesis” (ko00941) became the only two pathways whose rich factors were both larger than 0.05. The down-regulated DEGs were significantly enriched in “protein processing in endoplasmic reticulum” (ko04141), “starch and sucrose metabolism” (ko00500), “circadian rhythm – plant” (ko04712) and “vitamin B6 metabolism” (ko00750), while the up-regulated DEGs only significantly enriched in “flavonoid biosynthesis” (ko00941) with a corrected *p*-value of 3E-4 (Supplementary Figures S7a,b). However, there were no pathways significantly enriched in HO vs. LO (Figure 3B) when all DEGs were pooled together; nevertheless “Aminoacyl-tRNA biosynthesis” (ko00970) occupied the top degree enrichment with a corrected *p*-value of 0.083. Six DEGs were significantly up-regulated in “plant hormone signal transduction” (ko04075) (Supplementary Figures S7c,d).

For LO vs. LY, “Aminoacyl-tRNA biosynthesis” (ko00970), “Alanine, aspartate and glutamate metabolism” (ko00250) and “Glycine, serine and threonine metabolism” (ko00260) were the most enriched pathways with a corrected *p*-value between 0.05 and 0.1 (Figure 3C). Separate classifications (Supplementary Figures S7e,f) showed that the up-regulated DEGs were significantly enriched in “porphyrin and chlorophyll metabolism” (ko00860), “alanine, aspartate, and glutamate metabolism” (ko00250), “aminoacyl-tRNA biosynthesis” (ko00970) and “photosynthesis – antenna proteins” (ko00196). And “pathways – endocytosis” (ko04144), “fatty acid elongation” (ko00062), “ubiquitin mediated proteolysis” (ko04120), “phenylalanine metabolism” (ko00360) and “plant hormone signal transduction” (ko04075) were observed significant enrichment in down-regulated DEGs. Due to scant total DEGs (Figure 3D), as well as up- and down-regulated DEGs separately (Supplementary Figures S7g,h), no one pathway was enriched to a large extent in HO vs. HY. Only “vitamin B6 metabolism” (ko00750) became the most enriched pathways with a corrected *p*-value of 0.094 when total DEGs were compared.

In brief, *Ginkgo* trees of low age showed the disturbance of benzene propane, flavonoid biosynthesis, and circadian rhythm in the face of elevation rise. Flavonoid biosynthesis showed a significant up-regulation at elevated altitude. *Ginkgo* individuals of different ages at low altitudes show major differences in amino acid synthesis and metabolism. When they became older, significantly up-regulated unigenes are associated with chlorophyll and photosynthesis. Also, fatty acid elongation, phenylpropanoid synthesis, and plant hormone signal transduction were downgraded to varying degrees.

### Key Genes Involved in Flavonoid Biosynthesis Were Significantly Up-Regulated With an Elevation Increase and Fluctuated With an Age Increase

A total of 136 unigenes were putatively annotated in the flavonoid biosynthesis, including 5 naringenin 3-dioxygenase (F3H) genes, 15 *trans*-cinnamate 4-monooxygenase genes, 10 caffeoyl-CoA *O*-methyltransferase genes, 9 chalcone synthase (CHS) genes, 7 chalcone isomerase (CHI) genes, 5 anthocyanidin synthase (ANS) genes, 10 flavonol synthase (FLS) genes, 9 flavonoid 3′-monooxygenase (F3M) genes, 18 anthocyanidin reductase (ANR) genes, 4 coumaroylquinate (coumaroylshikimate) 3′-monooxygenase (C3′H) genes, 16 shikimate *O*-hydroxycinnamoyltransferase (HCT) genes, 10 leucoanthocyanidin reductase (LAR) genes, 17 bifunctional dihydroflavonol 4-reductase/flavanone 4-reductase (DFR) genes and 1 flavonoid 3′,5′-hydroxylase (F3′5′H) gene.

**FIGURE 4:**
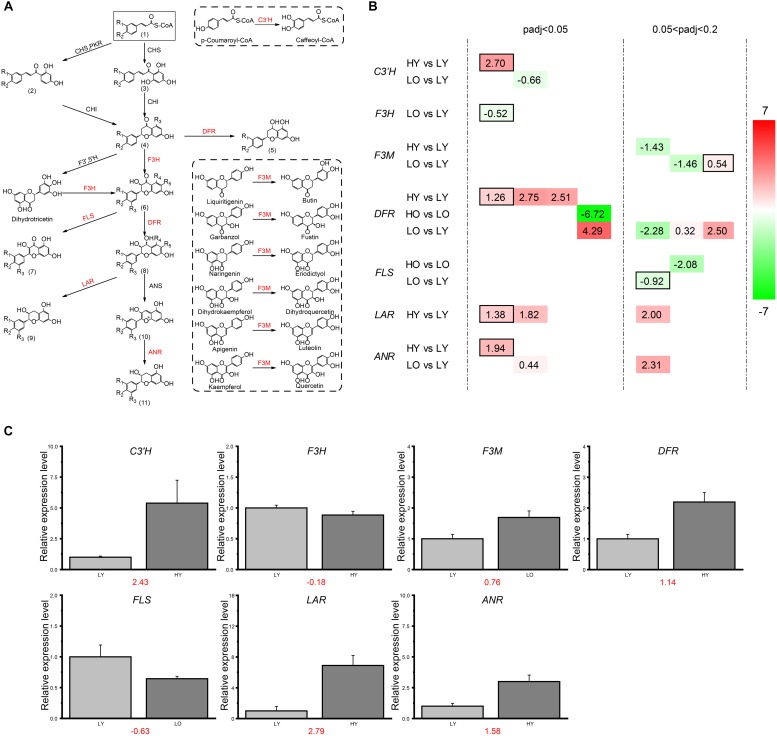
DEGs involved in the flavonoid biosynthesis. **(A)** The sketch map of the flavonoid biosynthesis pathway. In the flavonoid biosynthesis pathway, the functional enzymes corresponding to the genes screened and defined as DEGs in **(B)** are marked in red here. R in the chemical structure represents a hydrogen atom or a hydroxyl. (1) Cinnamoyl-CoA; *p*-Coumaroyl-CoA; Caffeoyl-CoA; (2) Isoliquiritigenin; Butein; (3) Pinocembrin chalcone; Naringenin chalcone; Eriodictyol chalcone; (4) Pinocembrin; Liquiritigenin; Butin; Naringenin; Eriodictyol; (5) Apiforol; Luteoforol; (6) Pinobanksin; Garbanzol; Fustin; Dihydrokaempferol; Dihydroquercetin; Dihydromyricetin; (7) Galangin; Kaempferol; Quercetin; Myricetin; (8) 5-Deoxyleucopelargonidin; 5-Deoxyleucocyanidin; *cis*-3, 4-Leucopelargonidin; Leucocyanidin; Leucodelphinidin; (9) Afzelechin; (+)-Catechin; (+)-Gallocatechin; (10) Pelargonidin; Cyanidin; Delphinidin; (11) (–)-Epiafzelechin; (–)-Epicatechin; (–)-Epigallocatechin. **(B)** DEGs involved in four comparisons. The different colors correspond to different expression levels with the log_2_(FC) value in each rectangle. For all putative genes, each column means the same unigene. **(C)** The qRT-PCR results of the key genes involved in flavonoid biosynthesis. The relative expression levels were presented in bar plot with the error bar by calculating the mean and stand deviation (SD) of cDNA copies in three independent replicates. The red numbers under the bar graphs indicated the log_2_(FC) values of each relative expression levels ratio in qRT-PCR. This result held an analogous trend of the transcriptome log_2_(FC) values of the corresponding putative unigenes which held the highest annotation similarity of each gene (black boxed in **B**).

Based on the strict threshold of adjusted *p*-value < 0.05 described previously, there were only 11 unigenes observed up- or down-regulation in four comparisons belonging to the flavonoid biosynthesis pathway (Figures 4A,B). So, to further investigate the expression level changes of flavonoid biosynthesis-related genes in different comparisons, the adjusted *p*-value was relaxed to 0.2. For HY vs. LY, one putative *C3*′*H* unigene, three putative *DFR* unigenes, three putative *LAR* unigenes and one putative *ANR* unigene were observed significantly up-regulated with the increase of elevation in the relatively younger age while only one putative *F3M* unigene was down-regulated. All these fold-change (FC) ratios were larger than 2 (|log_2_(FC)| > 1), indicating relatively dramatic variations toward these unigenes. However, in HO vs. LO, there were only one putative *DFR* unigene up-regulated with a log_2_(FC) value of 6.72 and one putative *FLS* unigene down-regulated with a log_2_(FC) value of −2.08. For LO vs. LY, one putative *F3M* unigene, three putative *DFR* unigenes and two putative *ANR* unigenes were identified different-level up-regulation when the plant age became older. Meanwhile, one putative *C3*′*H* unigene, one putative *F3H* unigene, one putative *F3M* unigene, one putative *DFR* unigene and one putative *FLS* unigene were down-regulated despite several |log_2_(FC)| values were less than one. Unfortunately, no DEGs were filtered in flavonoid biosynthesis in HO vs. HY.

In summary, a higher elevation led to more unigenes up-regulated than down-regulated in flavonoid biosynthesis in HY vs. LY at a young plant age level. However, when plant age became older in HO vs. LO, only one putative *DFR* unigene and one putative *FLS* unigene were both down-regulated with the increase of elevation. The situation was much more complicated when the influence of plant age was considered in LO vs. LY at a low elevation level. Two putative unigenes of *ANR* was observed up-regulated, and the filtered putative unigenes of *C3*′*H*, *F3H*, and *FLS* were all down-regulated with the decrease of plant age. Additionally, *F3M* possessed one up-regulated and one down-regulated putative unigenes. For *DFR*, three putative unigenes were up-regulated and one was down-regulated as plant age increased. Unexpectedly, no unigenes related to flavonoid biosynthesis were found significant variation when elevation increased in HO vs. HY. Seemingly, the flavonoid biosynthesis pathway was more sensitive and easier to be influenced when wild *Ginkgo* individuals were at low elevation or young age level. *DFR*s may be the key regulatory point for flavonoid biosynthesis disturbance in *Ginkgo* leaves.

### RT-qPCR Validation

RT-qPCR was used to verify the expression data of RNA-Seq and the subsequent splicing, assembly, and expression calculation. To exclude the influence of alternative splicing of gene unigenes, primers were designed in the homology regions of the unigenes which were transcribed from the same gene and retained high-similarity annotation. The log_2_(FC) results (Figure 4C) indicated the similarity between the RT-qPCR and the transcriptome situation of the filtered DEGs which held the highest high-similarity annotation of each gene (black boxed in Figure 4B).

### Flavonoid Derivatives Seemed to Show an Upward Trend With an Elevation Increase

The overlapping typical total ion chromatograms (TICs) of six QC samples obtained from LC–MS in negative mode (Supplementary Figure S8) demonstrated the acceptable error during the whole data collection. Meanwhile, six extracted ions (Supplementary Figure S9 and Supplementary Tables S7-1, S7-2, S7-3) were chosen to assess the repeatability of sampling and processing, as well as the stability and linearity of detection system by relative standard deviations (RSDs). The RTs varied from 0.03–1.40% on repeatability while 0.02–1.69% on intra-day variability and 0.04–1.89% on inter-day variability. For peak area, RSDs presented 6.13–15.68, 1.06–3.38, and 7.43–16.83% on repeatability, intra-day variability and inter-day variability, respectively. The mass accuracy possessed the RSDs from 8.77E-5 to 2.52E-4%. The linearity results showed the reliability of the experimental system with all R^2^s more than 0.997.

**FIGURE 5:**
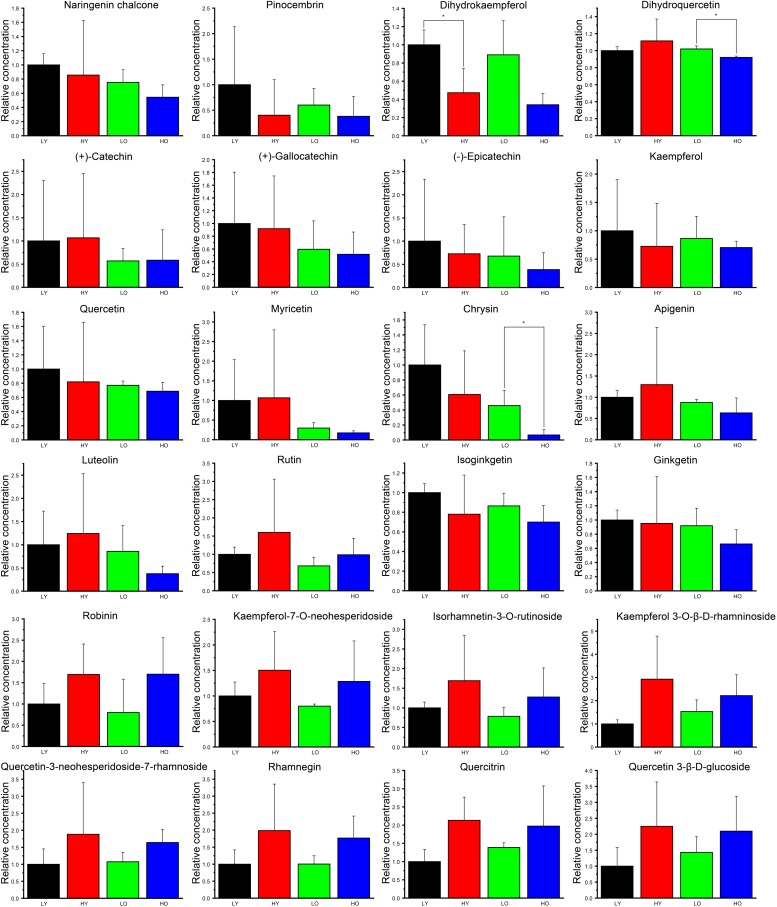
The relative concentration of identified flavonoids and derivatives. The black column indicates group LY. The red column indicates group HY. The green column indicates group LO. The blue column indicates group HO. Data are represented as the means ± SD of three biological replicates. Asterisks indicate significant differences (*p* < 0.01, Student’s *t*-test).

According to the non-targeted metabolites detection and identification by UPLC/qTOF MS method, A total of 13 ingredients involved in flavonoid biosynthesis pathway and 11 flavonoid downstream derivatives that possessed high parent ion signal intensity in MS were predicted and confirmed based on fragment information in multiple databases. The details of these ingredients, including RT, theoretical and experimental m/z, mass error and ions fragment information, were summarized in Table 1 and Supplementary Datasheet S3. The relative concentrations normalized from ion intensities were presented in the form of column charts with means and standard deviations in Figure 5. Unexpectedly, the differences in concentration of all ingredients between the four groups were not significant since *Ginkgo* trees grow in the wild.

**TABLE 1:** LC–MS identification results of metabolites related to flavonoid biosynthesis and flavonoid derivatives.

				**Adduct**	**Theoretical**	**Retention**	
**Compound name**	**CAS**	**Formula**	**Mass**	**type**	**m/z**	**time (min)**	**Fragment information**
Andrographolide^*^	5508-58-7	C_20_H_30_O_5_	350.2093	M+FA-H	395.2075	11.11	331.19, 287.20, 303.20, 239.18, 113.03
Naringenin chalcone	25515-46-2	C_15_H_12_O_5_	272.0685	M-H	271.0612	12.20	119.05, 151.01, 271.05, 107.02, 118.05
Pinocembrin	480-39-7	C_15_H_12_O_4_	256.0736	M-H	255.0663	15.06	255.07, 213.06, 201.84, 190.96, 151.00
Dihydrokaempferol	480-20-6	C_15_H_12_O_6_	288.0634	M-H	287.0561	9.94	125.03, 259.06, 177.06, 152.01, 201.06
Dihydroquercetin	480-18-2	C_15_H_12_O_7_	304.0583	M-H	303.0510	8.94	125.03, 175.04, 217.05, 199.04, 285.04
(+)-Catechin	154-23-4	C_15_H_14_O_6_	290.0790	M-H	289.0718	6.42	109.03, 203.07, 123.05, 226.06, 125.03
(+)-Gallocatechin	970-73-0	C_15_H_14_O_7_	306.0740	M-H	305.0667	5.97	125.03, 137.03, 219.07, 167.04, 139.04
(−)-Epicatechin	490-46-0	C_15_H_14_O_6_	290.0790	M-H	289.0718	7.49	203.07, 221.08, 123.05, 109.03, 137.02
Kaempferol	520-18-3	C_15_H_10_O_6_	286.0477	M-H	285.0405	12.39	285.04, 239.03, 214.03, 286.04, 268.04
Quercetin	117-39-5	C_15_H_10_O_7_	302.0427	M-H	301.0354	11.20	151.01, 301.03, 121.03, 107.02, 179.00
Myricetin	529-44-2	C_15_H_10_O_8_	318.0376	M-H	317.0303	9.91	137.03, 317.04, 179.00, 151.00, 167.04
Chrysin	480-40-0	C_15_H_10_O_4_	254.0579	M-H	253.0506	14.81	253.05, 163.00, 254.06, 187.04, 199.84
Apigenin	520-36-5	C_15_H_10_O_5_	270.0528	M-H	269.0455	12.17	269.05, 117.04, 151.00, 149.02, 225.06
Luteolin	491-70-3	C_15_H_10_O_6_	286.0477	M-H	285.0405	11.14	285.04, 133.03, 199.04, 151.00, 217.05
Rutin	153-18-4	C_27_H_30_O_16_	610.1534	M-H	609.1461	8.60	609.14, 301.03, 300.03
Isoginkgetin	548-19-6	C_32_H_22_O_10_	566.1213	M-H	565.1140	16.25	565.11, 533.08, 389.06, 374.04
Ginkgetin	481-46-9	C_32_H_22_O_10_	566.1213	M-H	565.1140	16.50	565.11, 533.09, 389.07, 374.04
Robinin	301-19-9	C_33_H_40_O_19_	740.2164	M-H	739.2091	10.54	739.18, 593.15, 284.03, 285.04, 255.03
Kaempferol-7-*O*-neohesperidoside	17353-03-6	C_27_H_30_O_15_	594.1585	M-H	593.1512	9.12	593.15, 285.04, 284.03
Isorhamnetin-3-*O*-rutinoside	604-80-8	C_28_H_32_O_16_	624.1690	M-H	623.1618	9.24	623.16, 315.05, 314.04, 300.03, 299.02
Kaempferol 3-*O-*β-D-rhamninoside	83170-31-4	C_33_H_40_O_19_	740.2164	M-H	739.2068	8.41	739.21, 284.03, 255.03, 227.04
Quercetin-3-neohesperidoside-7-rhamnoside	161993-01-7	C_33_H_40_O_20_	756.2113	M-H	755.2040	8.03	755.20, 300.03, 301.03, 271.02
Rhamnegin	75183-90-3	C_34_H_42_O_20_	770.2269	M-H	769.2197	8.50	769.21, 314.00
Quercitrin	522-12-3	C_21_H_20_O_11_	448.1006	M-H	447.0933	9.41	284.03, 300.03, 301.04, 447.09, 255.03
Quercetin 3-β-D-glucoside	482-35-9	C_21_H_20_O_12_	464.0955	M-H	463.0882	8.83	300.03, 301.04, 463.09, 271.02, 255.03

^*^The internal standard.

Overall, 10 ingredients seemingly showed higher concentration in LY than that in other three groups, including naringenin chalcone, pinocembrin, dihydrokaempferol, (+)-gallocatechin, (−)-epicatechin, kaempferol, quercetin, chrysin isoginkgetin and ginkgetin, although all fold changes were not large. Dihydrokaempferol was significantly less abundant in HY than that in LY. Dihydroquercetin and chrysin were both significantly less affluent in HO than LO. Irrespective of significance, the average contents of apigenin and luteolin were likely richer in HY than that in LY. Apart from isoginkgetin and ginkgetin, all other flavonoid downstream derivatives (rutin, robinin, kaempferol-7-*O*-neohesperidoside, isorhamnetin-3-*O*-rutinoside, kaempferol 3-*O*-β-D-rhamninoside, quercetin-3-neohesperidoside-7-rhamnoside, rhamnegin, quercitrin and quercetin 3-β-D-glucoside) were seemingly more abundant both in HY than in LY and in HO than in LO, which indicated that the increase of elevation may likely lead to the rise of the total flavonoid content. Simultaneously, 17 ingredients (naringenin chalcone, pinocembrin, dihydrokaempferol, (+)-catechin, (+)-gallocatechin, (−)-epicatechin, kaempferol, quercetin, myricetin, chrysin, apigenin, luteolin, rutin, isoginkgetin, ginkgetin, kaempferol-7-*O*-neohesperidoside, isorhamnetin-3-*O*-rutinoside) were more abundant both in LY than in LO and in HY than in HO. What’ more, a similar trend was observed in the comparison HO vs. HY about kaempferol 3-*O*-β-D-rhamninoside, quercetin-3-neohesperidoside-7-rhamnoside, rhamnegin, quercitrin and quercetin 3-β-D-glucoside. These signs suggested that an increase in plant age was more likely to result in a decrease in total flavonoids.

### Gene Fluctuations Led to an Accumulation of Lignin Monomers

**FIGURE 6:**
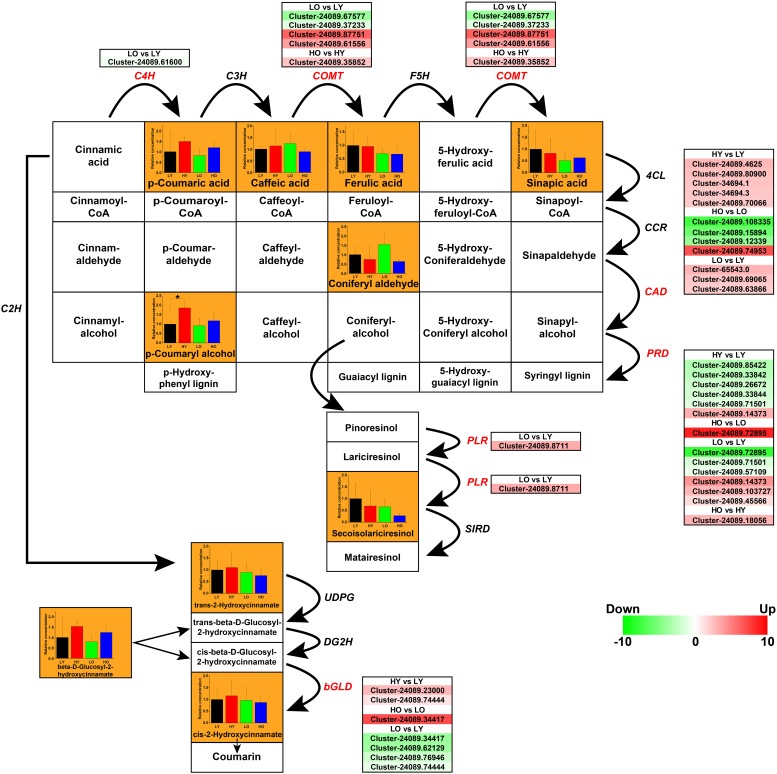
The transcriptomic and metabolic changes of non-flavonoids polyphenolics in the phenylpropanoid biosynthesis. The network of metabolite reactions was rebuilt in the form of a rectangular matrix, containing the names of various substances; the bold black arrows indicated the directions of metabolite reactions. The bar plots exhibiting the relative concentration of the ingredients identified in four comparisons were included in the corresponding rectangles (black LY, red HY, green LO, blue HO. ^*^p < 0.05, Student’s *t*-test). Each italic abbreviation corresponded to the putative gene that encoded the functional enzyme. The significantly up-regulated (red) and down-regulated (green) unigenes were listed beside the DEGs (red italic abbreviations).

The phenylpropanoid biosynthesis mainly includes the biosynthesis of HCAs, flavonoids, lignins, and lignans (Vogt, 2010). Main ingredients and genes involved in the biosynthesis of non-flavonoids polyphenolics were rebuilt in Figure 6. A total of 11 ingredients were confirmed based on UPLC-MS in Supplementary Table S8 and Supplementary Datasheet S3. 35 putative unigenes of C4H, caffeic acid 3-*O*-methyltransferase (COMT), cinnamyl-alcohol dehydrogenase (CAD), peroxidase (PRD), beta-glucosidase (bGLD), and pinoresinol/lariciresinol reductase (PLR) were observed visibly variability of gene expression in different comparisons (Figure 6).

*Trans-*cinnamic acid 2-hydroxylase (C2H), C4H, C3H, COMT, and F5H were the key enzyme in the process of converting cinnamic acid into HCAs. One putative *C4H* unigene and five putative *COMT* unigenes were observed different levels of fluctuation. However, the concentration statistics of *p*-Coumaric acid, Caffeic acid, Ferulic acid, and Sinapic acid indicated no significant variation between four comparisons. Seven putative *bGLD* unigenes were screened with notable differential gene expressions, which indicated that the rise of elevation might promote the conversion from *cis*-beta-D-Glucosyl-2-hydroxycinnamate to *cis-*2-Hydroxycinnamate while an older plant age suggesting a negative regulation on this process. Nevertheless, related metabolite detection is not significant enough to support this conclusion.

Noteworthily, five up-regulated *CAD* unigenes and five down-regulated *PRD* unigenes may be the main cause of a marked accumulation of *p*-Coumaryl alcohol when the elevation rose in HY vs. LY. They also have the potential possibility to promote the production of alcohols such as Caffeyl alcohol, Coniferyl alcohol, etc., although these metabolites not been identified. In this case, the up-regulation of *CAD*s and the down-regulation of *PRD*s may lead to the inhibition of lignins (Guaiacyl lignin, Syringyl lignin, etc.) biosynthesis, which is more conducive to the synthesis of lignans. However, there was no significant evidence of *PLR*s and *SIRD*s, as well as the only concentration analysis of Secoisolariciresinol to support this speculation. When the age of *Ginkgo* trees increased in LO vs. LY, although some putative unigenes of *CAD*, *PRD*, and *PLR* showed an upward trend, the similarity of functional annotations was not high. In addition, there was no indication that the concentrations of *p*-Coumaryl and Secoisolariciresinol have increased significantly.

Hence, the elevation increase may lead to the accumulation of lignin monomers, like 4-Coumaryl alcohol, Coniferyl alcohol, 5-Hydroxyconiferyl alcohol and Sinapyl alcohol, but inhibit the lignification by up-regulating *CAD*s and down-regulating *PRD*s. Thus, a hypothesis was presumed that the rise of elevation might potentially inhibit the synthesis of lignins, leading to an accumulation of Caffeyl alcohol, Coniferyl alcohol, etc. HCAs and lignans showed no obvious variational trend in this situation. Age variability caused related genetic perturbations, but there was insufficient evidence to prove the specific impact.

## Discussion

### Light Induction May Be the Main Reason for Flavonoids Accumulation in the Case of Elevation Rise

Flavonoids have long been recognized as the primary antioxidants in plants which are useful in preventing UV-B radiation from penetrating into sensitive tissues and protecting against programmed cell death mediated by oxidative stress (Sadik et al., 2003; Michalak, 2006; Agati and Tattini, 2010; Agati et al., 2013). The rise of elevation directly leads to higher exposure to UV radiation and lower temperature, inducing the production of flavonoids (Jugran et al., 2016). However, the flavonols appear to accumulate with the light enhancement, while be little affected by temperature (Jaakola and Hohtola, 2010). In this research, an approximate 400 m increase of elevation in HY vs. LY could not cause a visible decrease in temperature, meanwhile implying that the excess light and the higher UV radiation may be the main reason for the accumulation of flavonoids. Mainly in the form of glycosides, the identified flavonoids derivatives (viz. Robinin, Quercitrin, Rhamnegin, etc.) presented a potentially positive correlation to the elevation increase, which seems to be inconsistent with the existing theories that high light irradiance accelerates the biosynthesis of dihydroxy B-ring-substituted flavonoids, but does not affect the biosynthesis of monohydroxy B-ring-substituted flavonoids (Ryan et al., 1998; Agati et al., 2011). Understandably, although B-ring dihydroxylated flavonoids display higher antioxidant activity toward ROS, the monohydroxylated ones exert a higher capacity of UV wavelengths absorption (Nabavi et al., 2018). In addition, the increase of elevation may cause an enhancement of other abiotic stresses, which may be the possible interpretation of an up-regulated tendency of some monohydroxy B-ring-substituted flavonoids, like Apigenin and Kaempferol. Notably, it has been confirmed that the synthesis of flavonoids is closely related to the regulation of auxin and abscisic acid (ABA) (Brunetti et al., 2018; Kumar et al., 2018). The ABA, reported to promote the accumulation of flavonols (Berli et al., 2010, 2011), was observed to have an active integration with the light signaling (Bechtold et al., 2008; Wang et al., 2018), which may help to explain the enhancement of flavonol biosynthesis in leaves exposed to high light irradiance, even without ultraviolet radiation (Brunetti et al., 2018).

### DFR May Be the Key Regulatory Point for Light-Induced Flavonoid Biosynthesis Disturbance in *Ginkgo* Leaves

Numerous evidence suggested that more than one homologous *DFR* unigenes existed in multiple plant species (Beld et al., 1989; Tanaka et al., 1995; Cassetta et al., 2013; He et al., 2015). Under light-induced conditions, the expression of *DFR*s was confirmed to change significantly, leading to the accumulation of flavonoid anthocyanins, condensed tannins and other substances (Paolocci et al., 2005; Shi et al., 2014). During flower color development, *DFR*s exhibited distinctive variability in circadian rhythms of *Anthurium andraeanum*, indicating its potentially feature as a critical regulatory point of anthocyanins biosynthesis (Collette et al., 2004). However, in the green leaves, especially of a woody plant, the potential regulatory status of *DFR* has not been fully discussed yet. Three *DFR* cDNA clones were isolated from *Ginkgo biloba*, of which *GbDFR2* and *GbDFR3* were related to anthocyanin accumulation in leaves while *GbDFR1* seemed to be involved in environmental stress response (Cheng et al., 2013). In this research, the gene expression profile seemed to support a view that *DFR*s may be the key regulatory point for light-induced flavonoid biosynthesis disturbance in *Ginkgo* leaves. The expression profiles of DEGs (Figure 4B) involved in flavonoid biosynthesis and the amino acid-based phylogenetic trees (Supplementary Figure S10) of related unigenes suggested the view that DFR disturbances were significantly stronger and more sensitive than other flavonoid synthesis-related genes when facing to the change of the elevation and plant age. The *DFR*s of high annotation similarity, especially Cluster-24089.39479 and Cluster-24089.54759 may potentially play a major role in the accumulation of antioxidants under light induction. Moreover, in the comparison of HY vs. LY, the significantly up-regulated *LAR* and *ANR* with the rise of various flavonoid glycosides may also be a good illustration of the effects of illumination enhancement. Although many cases indicated that *CHS* might produce significant responses to the UV stimulation (Chappell and Hahlbrock, 1984; Batschauer et al., 1996), no *CHS* unigenes were found to be notably altered in this study. Most of the researches in this filed were based on herbaceous plants, while woody plants need more comprehensive and in-depth insights to unveil the potential metabolic properties. In addition, *DFR*s may be more sensitive to the weaker changes in light intensity caused by small elevation variability in *Ginkgo*.

### The Age Effects of *Ginkgo* Trees on the Biosynthesis of Flavonoids

Nowadays, the temporal effects on the flavonoid biosynthesis were mainly focused on seasonality, circadian rhythm and the growth stages of leaves, flowers or fruits in herbs (Zhang et al., 2003; Collette et al., 2004; Nakatsuka et al., 2005; Crecelius et al., 2017), whilst only a few discussions were carried out on the age effects of the perennial woody plant, especially some longevity species like *Ginkgo biloba* and *Picea abies*. An accumulation of flavan-3-ols (especially proanthocyanidin and (−)-epicatechin) has been reported to be simultaneous with the age increase of *Cistus clusii*, a perennial evergreen bush (Hernández et al., 2011). However, in this study, no significant variability of (−)-epicatechin was observed with the age increase of the *Ginkgo* trees (LO vs. LY in Figure 5). Moreover, although the age increase caused disturbances in several genes’ expression in flavonoid biosynthesis, such as the up-regulated *DFR*s and *ANR*s (Figure 4B), the relative concentration of most identified flavonoids or flavonols indicated a declining tendency of no statistically significance with the increase of *Ginkgo* age (LO vs. LY). On the other hand, although the senescence process has been reported to be correlated with the accumulation of phenolics in *Pinus* needles (Szymura, 2009; Kumar et al., 2018), no similar indication was observed existence in this study. Until now, no detailed discussions were taken out on the signs of *Ginkgo* senescence. Therefore, whether flavonoids play a vital role in the senescence of *Gingko* individuals requires further research.

### The Transcriptomic and Metabolic Influence on Non-flavonoids Polyphenolics in the Phenylpropanoid Biosynthesis

There was no significant correlation between HCAs and elevation increase in this study. Increased altitude means increased illumination. The HCAs changes under light induction have long been questioned. Excessive sunlight exposure led to a decrease of HCAs:flavonoid ratio in *Ligustrum vulgare* leaves (Tattini et al., 2004), which held a similar ratio trend to this study. Inconsistently, a decrease of HCAs content was observed with the light enhancement in the roots of *Arabidopsis* (Hemm et al., 2004). However, high visible radiation could effectively stimulate the synthesis of hydroxycinnamic acid in grape leaves (Kolb et al., 2001). Different results may result from different species and tissues for sampling. The limited increase in average light intensity caused by elevation increase may be the potential reason why no significant changes have been observed in HCAs content.

Lignin biosynthesis, as a highly energy-consuming and irreversible process, responds to many environmental factors like light, sugar content, biological clocks (Zhao and Dixon, 2011). It has been reported that high-light stress could enhance lignin synthesis in *Arabidopsis* seedlings (Kimura et al., 2003) and hexose carbohydrates, the photosynthesis products, could promote lignin synthesis at night, relying on circadian rhythms (Rogers et al., 2005). However, in *Ginkgo* leaves, no significant signs were observed to reflect the light influence on lignin synthesis. The light-induced enhancement of lignin biosynthesis may be not fully applicable to the leaves of mature woody plants. Lignin biosynthesis genes are preferentially expressed in actively lignifying tissues, while many lignin repressors are preferentially expressed in non-lignifying tissues (Zhao and Dixon, 2011). For mature *Ginkgo* individuals, the lignification degree of the trunk and branches always are much higher than that of the leaves, so the expression of the lignification gene should be mainly concentrated in the branches rather than the leaves. This may also be the potential reason why the age variability of *Ginkgo* trees had not caused significant changes in the lignin synthesis pathway in leaves in this study.

The illumination change caused by elevation rise showed insignificant disturbances for lignans biosynthesis in this study. CPI-FK cells were reported to show a positive response to white fluorescent, red LED and blue LED light in the synthesis of lignans, indicating that light enhancement has the potential to improve lignans production (Morimoto et al., 2011; Satake et al., 2015). However, this phenomenon has only been verified in *Forsythia koreana* and *Sesamum indicum*, and verifications of more species need to proceed further.

In summary, the weak illumination enhancement caused by the elevation change may be the main reason for the significant accumulation of lignin monomers. However, there were no significant changes in HCAs, lignins and lignans. The potential reason was the mild variability of environmental factors, unlike the artificial designs of dramatic environmental difference. In addition, different physiological status caused by age changes did not cause significant fluctuations in the biosynthesis of non-flavonoids polyphenolics. As few insights were thrown to study the influence of individual age on its tissue metabolism, more focus and discussions should be paid to this field.

### Flavonoid Biosynthesis May Be More Sensitive to Elevation and Tree Age Than Other Branch Pathways Involved in Phenylpropanoid Biosynthesis

An elevation increase of 300–400 m may result in a moderate enhancement in illumination intensity. In this situation, DEGs analysis seemed to show a stronger disturbance in flavonoid biosynthesis than in other non-flavonoids polyphenolics pathways, which indicated that flavonoid biosynthesis might be more sensitive to elevation and tree age than other branch pathways involved in phenylpropanoid biosynthesis. Metabolites profile seemed to support this conjecture. Otherwise, the synthesis of these polyphenolics is based on the same substrate, which will inevitably lead to the existence of competition. A light-induced metabolism analysis in *Arabidopsis* roots appeared to support this inference (Hemm et al., 2004).

## Conclusion

This research is the first insight into the effects of elevation and plant age on phenylpropanoid biosynthesis in wild *Ginkgo* trees, based on transcriptomics and metabolomics analysis. The key genes involved in flavonoid biosynthesis, including *DFR*, *LAR*, and *ANR* were significantly up-regulated with the elevation increase. Mass spectrometry analysis indicated a similar potential trend that the content of most flavonoid derivatives increased with the elevation rise. The light fluctuation caused by elevation increase may be the main impetus in this process. *DFR*s appeared to exhibit the most sensitive responses in the face of environmental changes; therefore, might be the main regulatory point in the flavonoid synthesis. Although the age variability only affected the expression of related genes to a lesser extent, metabolites analysis speculated that the growth of *Ginkgo* age might be detrimental to flavonoid synthesis in leaves. In addition, multiple genes implicated in the synthesis of HCAs, lignin, and lignan exhibited fluctuations with the elevation changes. Notably, significantly up-regulated *CAD*s and down-regulated *PRD*s potentially led to the accumulation of *p*-Coumaryl alcohol, one of the lignin monomers, and might inhibit further lignification, while the age increase did not observe a significant impact. Therefore, flavonoid biosynthesis exhibited a higher degree of perturbation than that of HCAs, lignins and lignans, potentially suggesting that flavonoid biosynthesis might be more susceptible than other branch pathways involved in phenylpropanoid biosynthesis. However, moderate environmental changes may mask fluctuations in insensitive components in this study. And the effect of *Ginkgo* age on the synthesis of phenylpropanoids in leaves needs further excavation. In conclusion, these findings can facilitate the functional genomic and metabolic research in *Ginkgo*, and provide a reference for artificial planting and industrial harvesting.

## Author Contributions

KZ, YL, QY, and XL conceived and designed the work. KZ, QY, SF, WC, and RL performed the experiments. KZ, DZ, WC, and RL analyzed the data. KZ wrote and revised the manuscript. All authors read and approved the final manuscript.

## Conflict of Interest Statement

WC was employed by the company Zhejiang CONBA Pharmaceutical, Co., Ltd., Hangzhou, China. The remaining authors declare that the research was conducted in the absence of any commercial or financial relationships that could be construed as a potential conflict of interest.

## Footnotes

1 http://www.genome.jp/kegg/kaas/

2 http://www.hmdb.ca/

3 https://pubchem.ncbi.nlm.nih.gov/

4 https://www.nist.gov/

5 http://www.massbank.jp/

6 https://metlin.scripps.edu/
