# Supplementary material for: Flavonoid Biosynthesis Is Likely More Susceptible to Elevation and Tree Age Than Other Branch Pathways Involved in Phenylpropanoid Biosynthesis in Ginkgo Leaves
Source: Front Plant Sci. 2019 Jul 30;10:983. doi: 10.3389/fpls.2019.00983 (PMC6682722; doi:10.3389/fpls.2019.00983)
Supplement: Supplementary file 1 [file Table_1.DOCX]

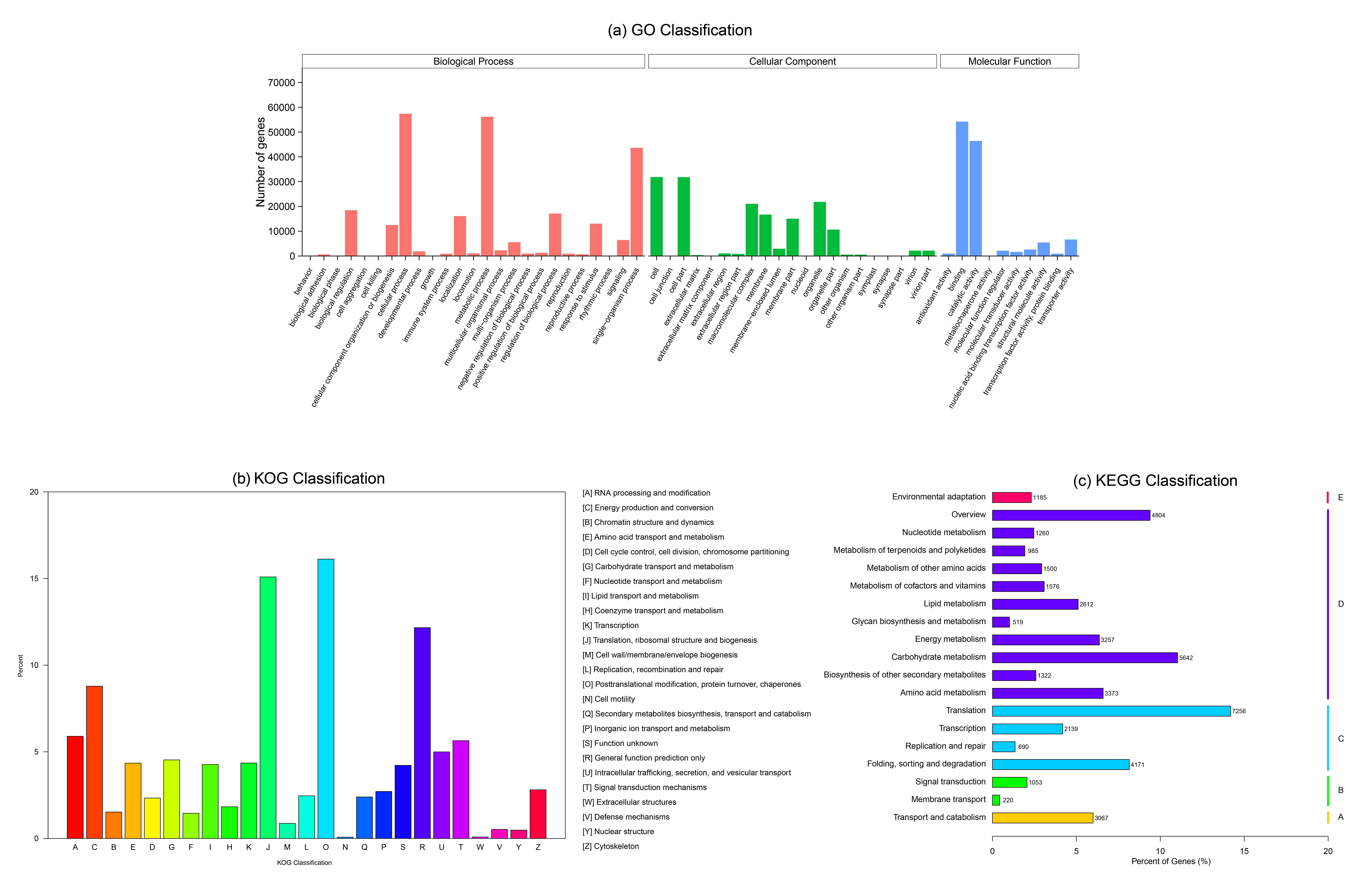


**Figure S1. The detailed functional classification of the annotated unigenes.** (**a**) GO classification (**b**) KOG classification (**c**) KEGG classification. A Cellular Processes, B Environmental Information Processing, C Genetic Information Processing, D Metabolism, and E Organismal Systems


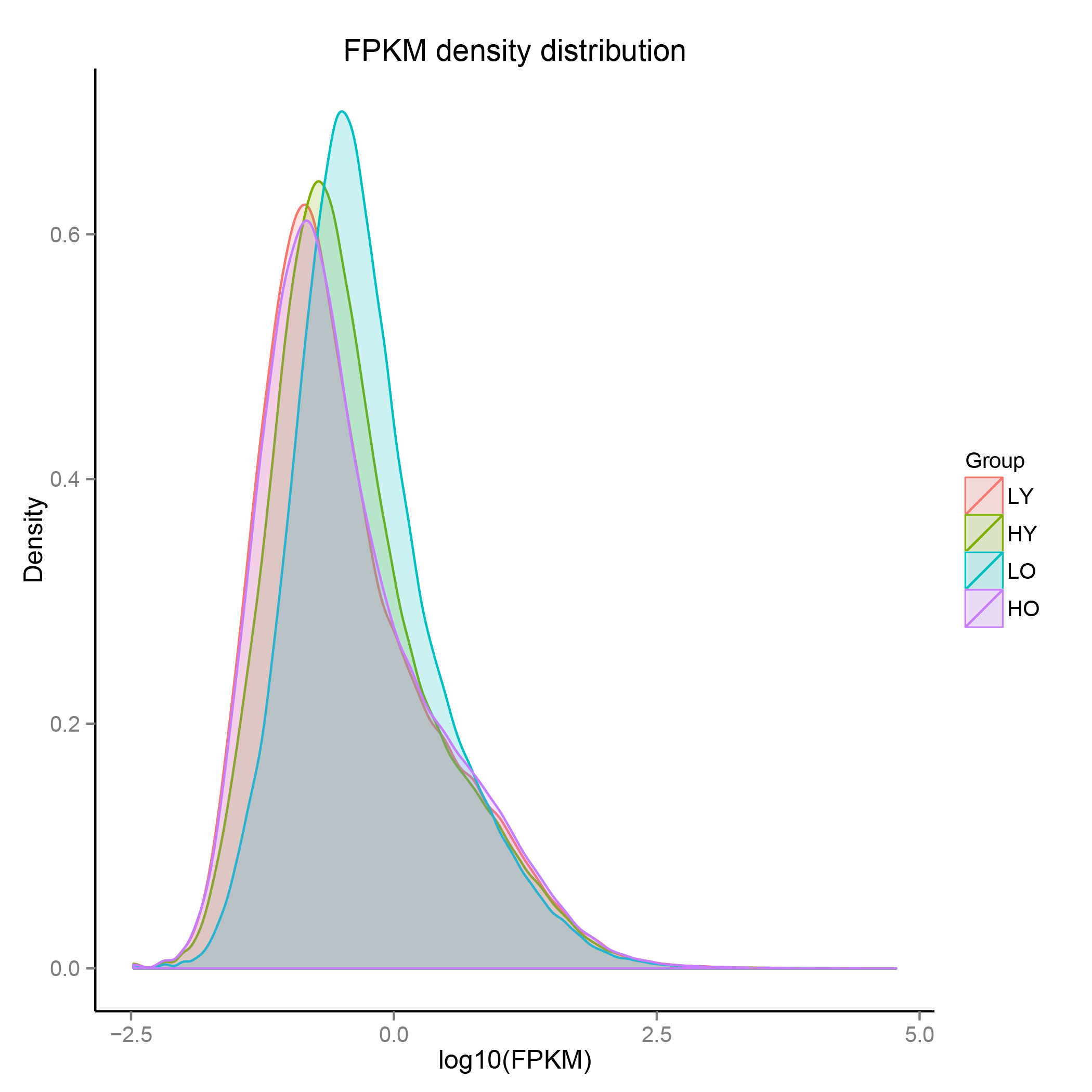


**Figure S2. Density distribution of gene FPKMs in each group**


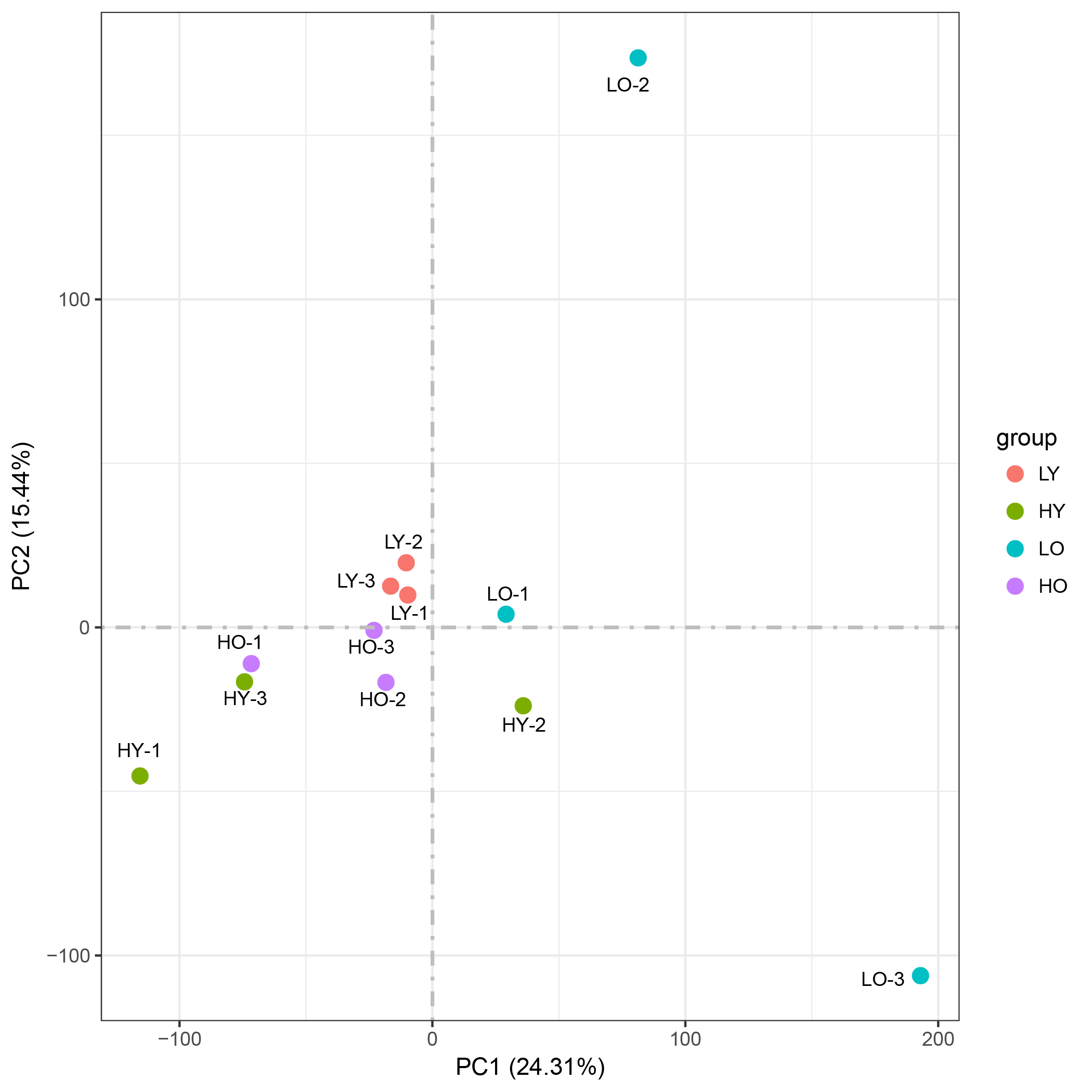


**Figure S3. PCA plot between four groups.**


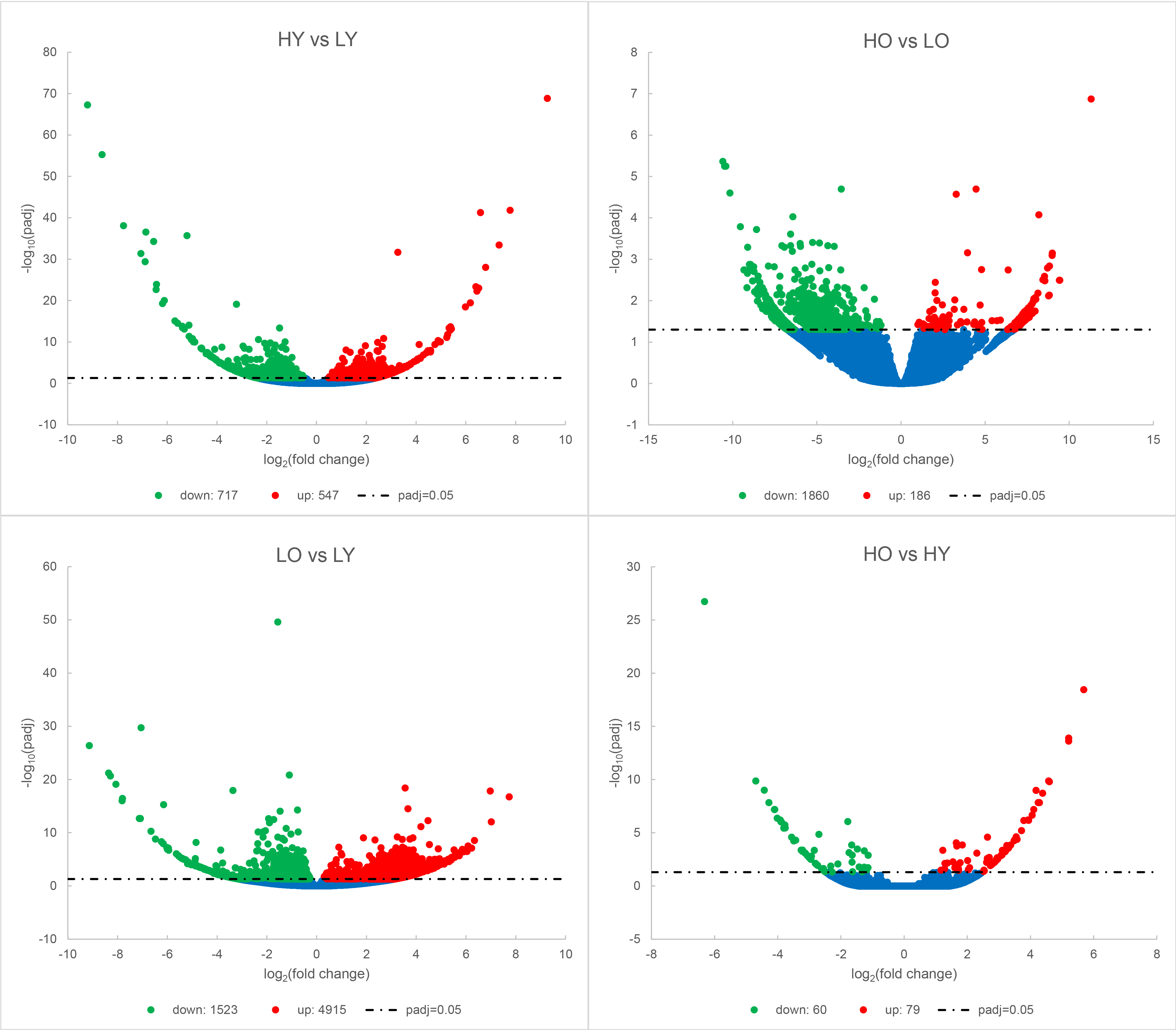


**Figure S4 Volcano plot of DEGs Filtering.** Red dots indicated significantly up-regulated unigenes. Green dots indicated the significantly down-regulated unigenes. Blue dots indicated the unigenes with no significant variation.


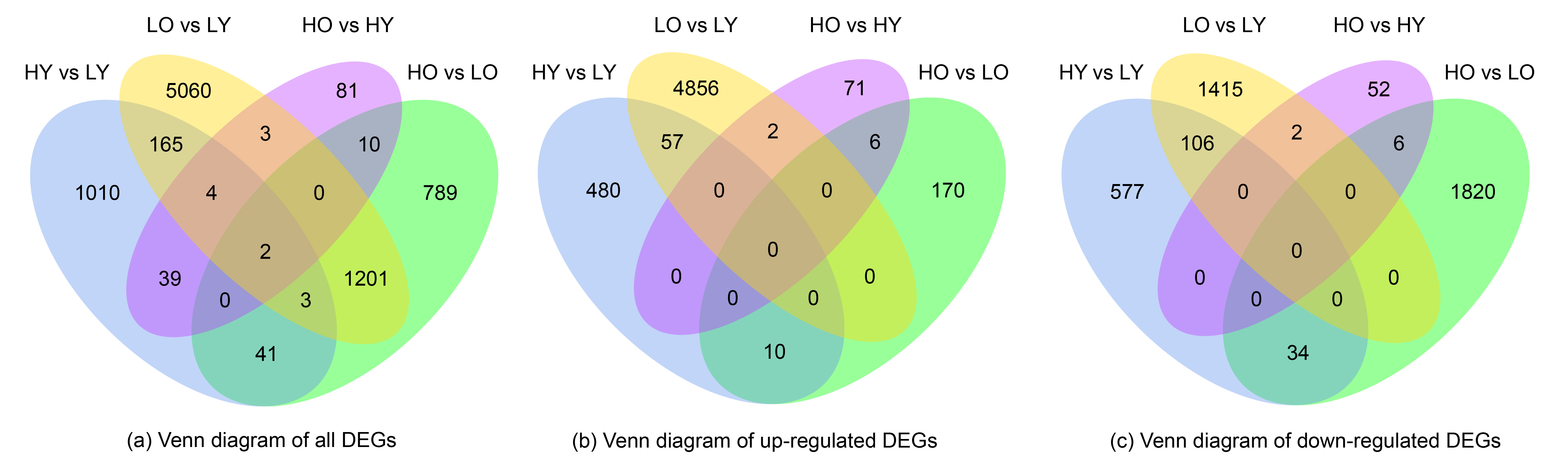


**Figure S5. Venn diagram of DEGs**

**
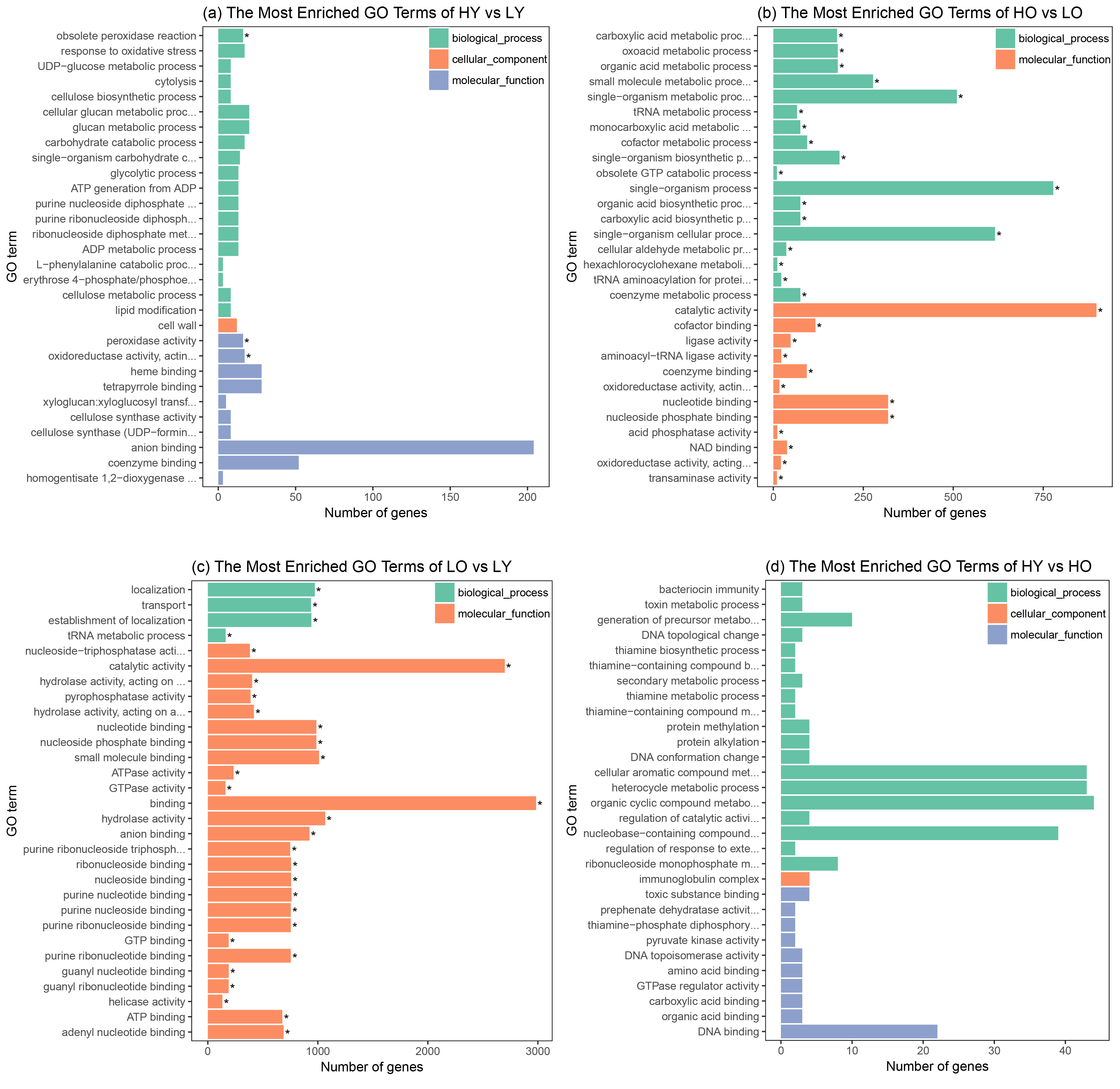
**

**Figure S6. GO enrichment of all comparisons.** (**a**) The most enriched GO terms of HY vs LY. (**b**) The most enriched GO terms of HO vs LO. (**c**) The most enriched GO terms of LO vs LY. (**d**) The most enriched GO terms of HY vs HO. The corrected P-value was used to assess the degree of enrichment. The GO terms with Corrected P-Value<0.05 were defined as the significant enriched ones, which were marked with asterisks.

**
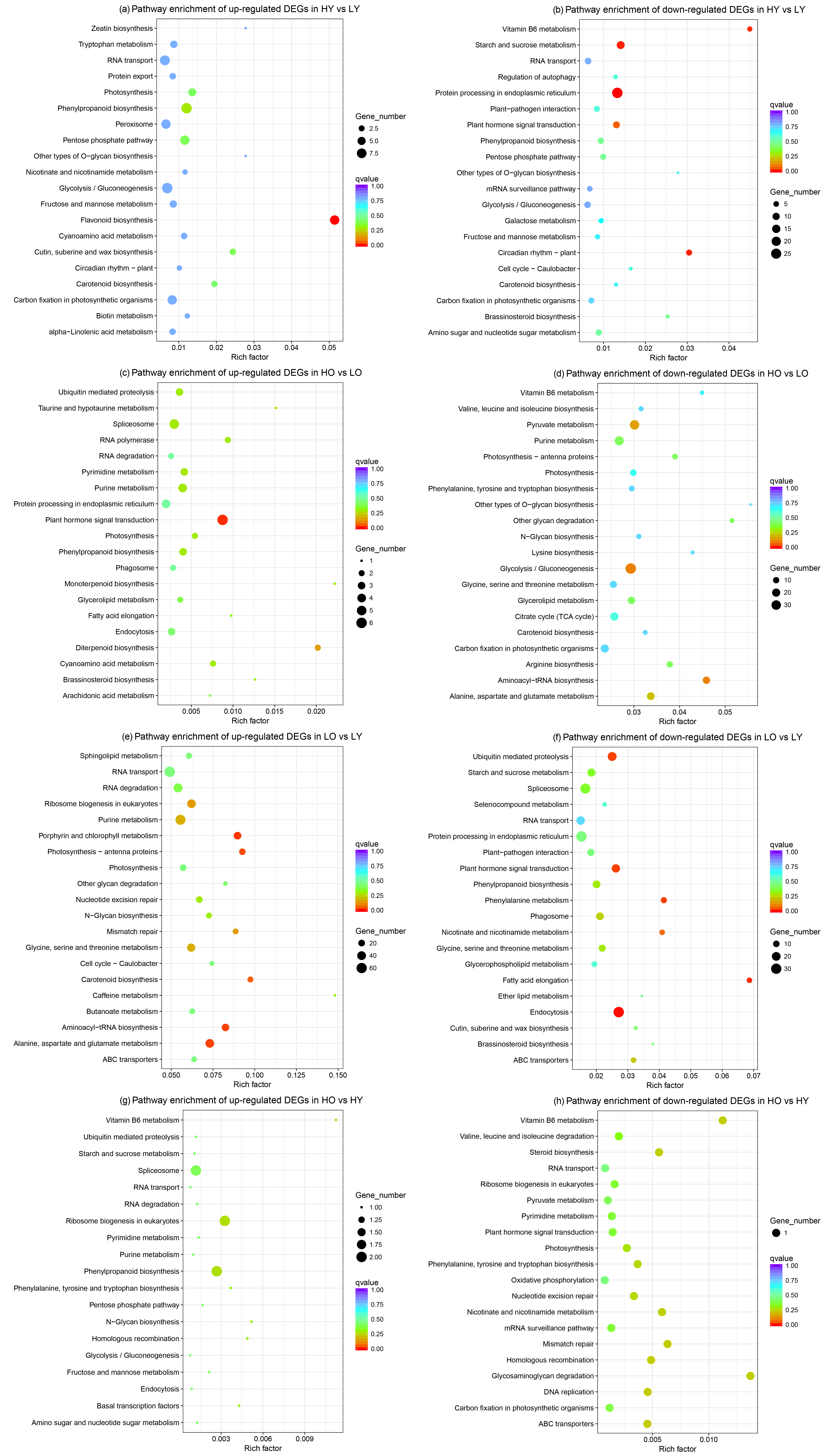
**

**Figure S7. KEGG pathway enrichment of up- and down-regulated DEGs of each comparison.** (**a**) The most enriched KEGG pathways of up-regulated DEGs in HY vs LY. (**b**) The most enriched KEGG pathways of down-regulated DEGs in HY vs LY. (**c**) The most enriched KEGG pathways of up-regulated DEGs in HO vs LO. (**d**) The most enriched KEGG pathways of down-regulated DEGs in HO vs LO. (**e**) The most enriched KEGG pathways of up-regulated DEGs in LO vs LY. (**f**) The most enriched KEGG pathways of down-regulated DEGs in LO vs LY. (**g**) The most enriched KEGG pathways of up-regulated DEGs in HO vs HY. (**h**) The most enriched KEGG pathways of down-regulated DEGs in HO vs HY.

**
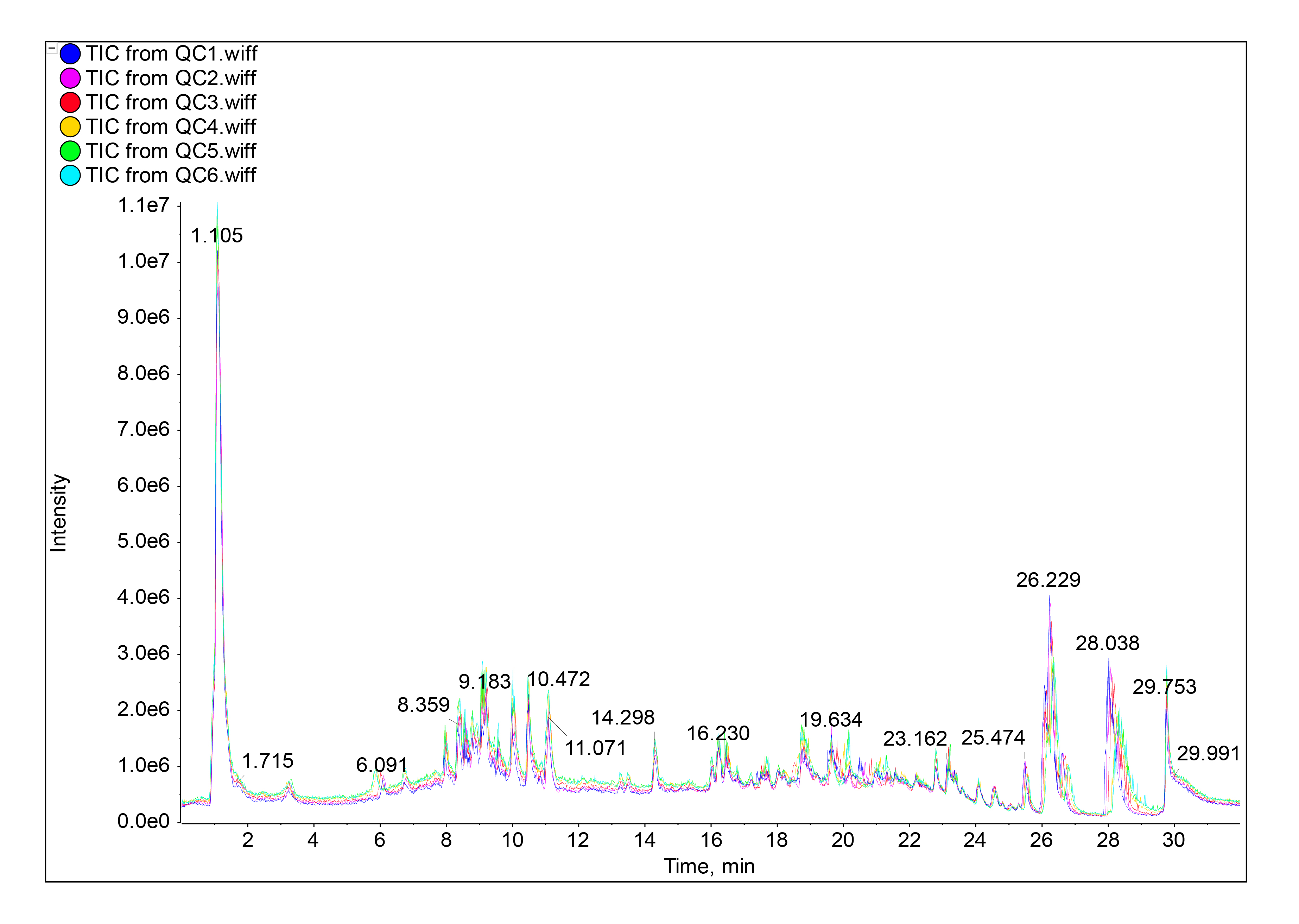
**

**Figure S8. The overlapping typical total ion chromatograms (TICs) of QC samples**

**
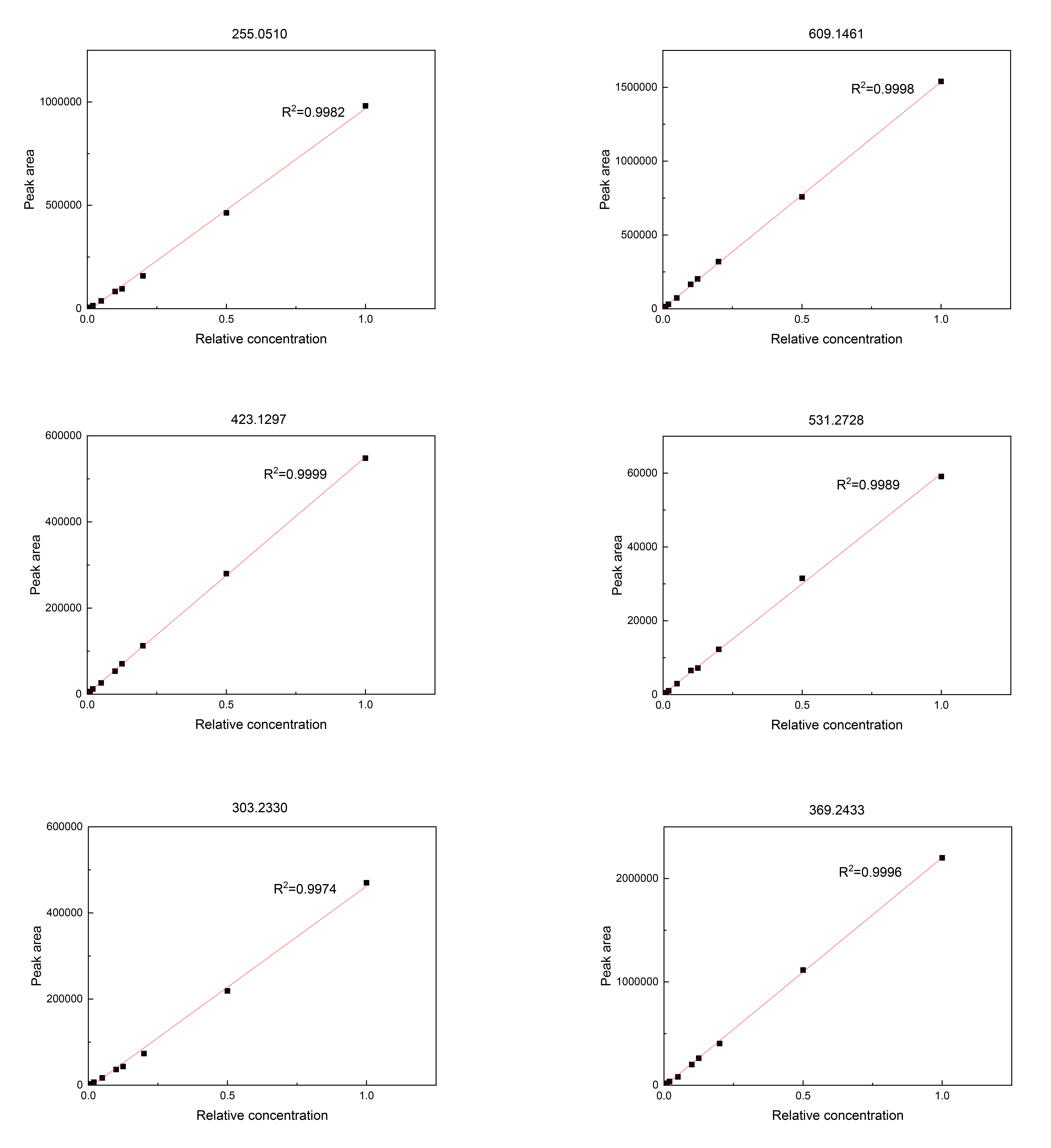
**

**Figure S9. The linearity of the methodological investigation.**

**
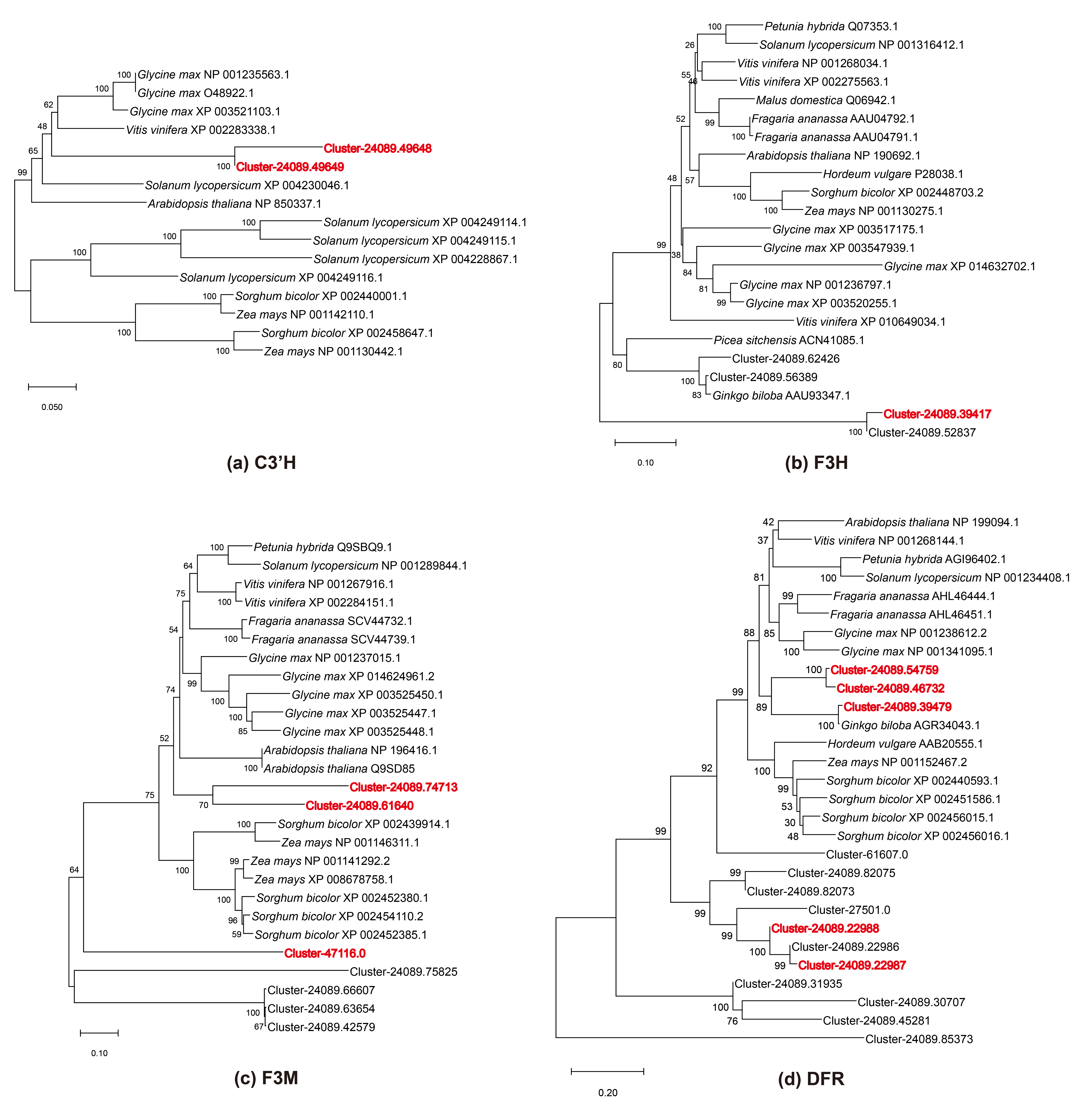

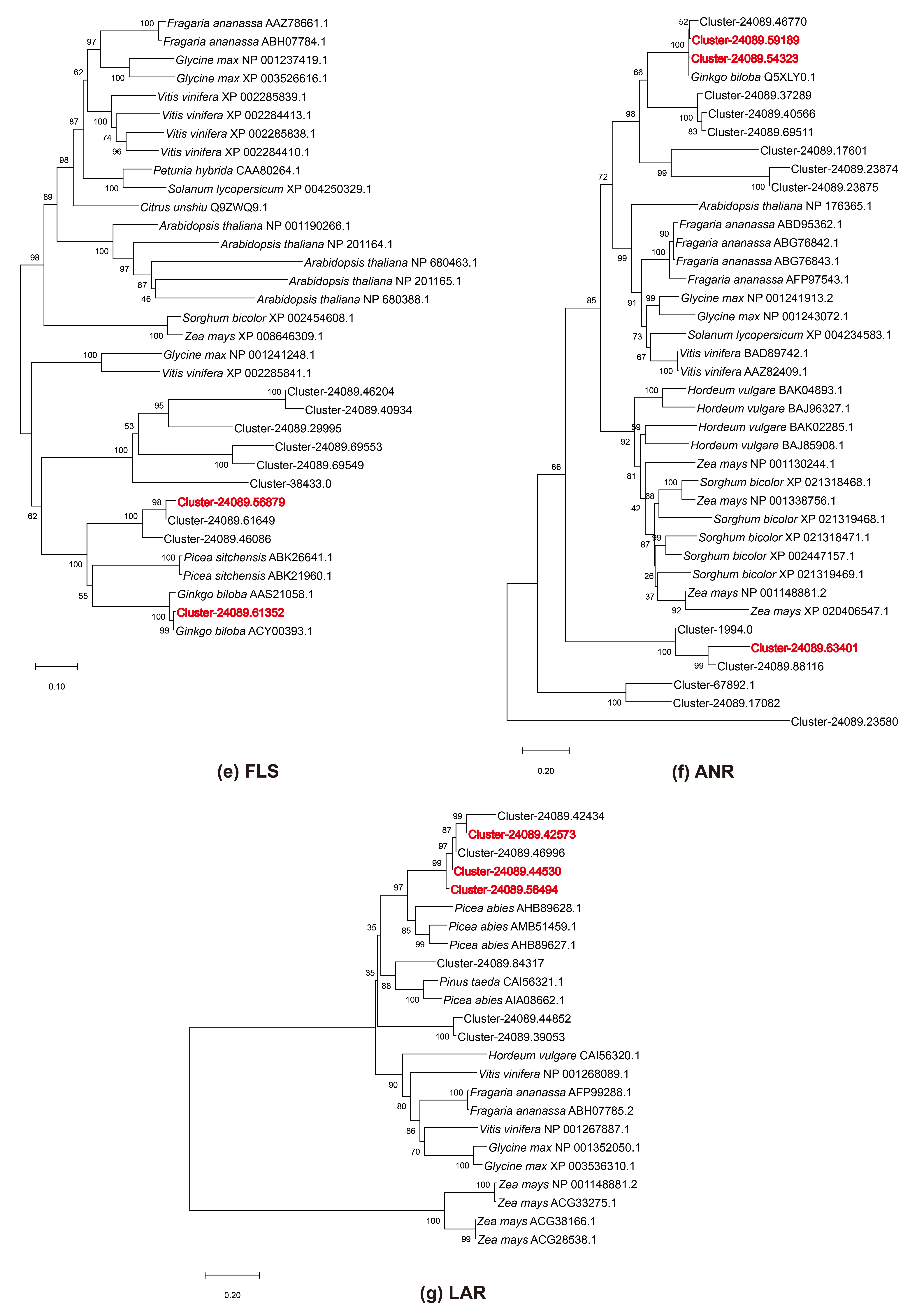
**

**Figure S10 Phylogenetic trees of plant DFRs based on amino acid sequences.** Multiple species were selected to produce phylogenetic trees of key enzymes in flavonoid synthesis. The trees were constructed by the Neighbor-Joining method, MEGA-X (version 10.0.5). The numbers at each node represented the bootstrap value, with 1000 replicates. The red labeled items means the corresponding DEGs.
