# Supplementary material for: Flavonoid Biosynthesis Is Likely More Susceptible to Elevation and Tree Age Than Other Branch Pathways Involved in Phenylpropanoid Biosynthesis in Ginkgo Leaves
Source: Front Plant Sci. 2019 Jul 30;10:983. doi: 10.3389/fpls.2019.00983 (PMC6682722; doi:10.3389/fpls.2019.00983)
Supplement: Supplementary file 2 [file Table_2.DOCX]

**Table S1. Twelve ginkgo trees used for sampling.**

| NO. | Group^1^ | Sample ID | DBH^2^ (m) | Elevation (m) | Latitude | Longitude | Sexuality |
| --- | --- | --- | --- | --- | --- | --- | --- |
| 1 | LY | LY1 | 0.43 | 800 | N 29°56'55" | E 110°39'16" | female |
| 2 |  | LY2 | 0.53 | 600 | N 29°57'41" | E 110°46'08" | male |
| 3 |  | LY3 | 0.75 | 650 | N 29°51'59" | E 110°44'59" | female |
| 4 | HY | HY1 | 0.57 | 1140 | N 30°6'34" | E 110°46'27" | male |
| 5 |  | HY2 | 0.61 | 1020 | N 29°55'50" | E 110°37'6" | female |
| 6 |  | HY3 | 0.72 | 1130 | N 30°6'44" | E 110°46'25" | female |
| 7 | LO | LO1 | 1.03 | 660 | N 29°51'59" | E 110°44'59" | female |
| 8 |  | LO2 | 1.27 | 530 | N 29°57'22" | E 110°48'40" | male |
| 9 |  | LO3 | 1.4 | 800 | N 29°56'55" | E 110°39'16" | female |
| 10 | HO | HO1 | 1.21 | 1160 | N 30°6'7" | E 110°49'2" | female |
| 11 |  | HO2 | 1.31 | 1040 | N 26°55'13" | E 110°36'16" | female |
| 12 |  | HO3 | 1.69 | 1000 | N 29°56'7" | E 110°38'28" | male |

^1^ All samples were divided into four groups - low elevation and young age (LY), high elevation and young age (HY), low elevation and older age (LO), high elevation and older age (HO).

^2^ DBH is referred to the diameter at breast height.

**Table S2. Primers used in RT-qPCR.**

| Gene | Forward (5'-3’) | Reverse (5'-3’) | Produce Size (bp) |
| --- | --- | --- | --- |
| *GAPDH* | TCCACGGGAGTCTTCACTGACA | TGCTCATTCACGCCAACAACGA | 79 |
| *C3'H* | GCACCCGCCAACTCCTTTGA | ACAGCCGGATCACGAGCAATG | 126 |
| *F3H* | CTTCGTCGTCAGCAGCCATCT | CGAGAGCGGAGAGGATAGGAGA | 87 |
| *F3M* | TCGTCACAGATAGCCGCTTGGT | TGTCTCCTTGGCAACGCAATCA | 77 |
| *DFR* | TGCCATTGACGGATGCGAAGG | TCTCTGGGTCCTGCGACTCAAA | 71 |
| *FLS* | GAGCAACGGCAAGTTCAAGAGC | CAGAATACCGGCCACGACATCC | 81 |
| *LAR* | TCTACGCTGGAGGAGGTGACAT | CGATCCTGGCATTCTCGGAGTT | 81 |
| *ANR* | GCAGAAGAGCACAGCCTTGACG | ACGCTAGACGGCACTGTTGGT | 86 |

**Table S3. Summary of sequencing result after filtering**

| Sample | RIN |  | Raw Reads | Clean Reads | Clean Bases | Error (%) | Q20(%) | Q30(%) | GC Content (%) |
| --- | --- | --- | --- | --- | --- | --- | --- | --- | --- |
| LY1 | 6.9 |  | 44854254 | 43147222 | 6.47G | 0.02 | 97.00 | 92.58 | 46.12 |
| LY2 | 6.6 |  | 46874430 | 45578352 | 6.84G | 0.02 | 96.62 | 91.39 | 46.55 |
| LY3 | 6.6 |  | 43578594 | 41883522 | 6.28G | 0.02 | 96.84 | 92.25 | 45.89 |
| HY1 | 7.0 |  | 48880520 | 48004270 | 7.20G | 0.01 | 97.46 | 93.52 | 46.89 |
| HY2 | 6.9 |  | 51250856 | 49320128 | 7.40G | 0.02 | 96.99 | 92.52 | 47.00 |
| HY3 | 7.4 |  | 53062588 | 52128974 | 7.82G | 0.01 | 97.55 | 93.72 | 46.26 |
| LO1 | 7.4 |  | 44692318 | 42914578 | 6.44G | 0.02 | 96.69 | 91.92 | 46.45 |
| LO2 | 7.2 |  | 54988838 | 53474142 | 8.02G | 0.02 | 96.59 | 91.37 | 46.75 |
| LO3 | 6.6 |  | 52757564 | 50699804 | 7.60G | 0.02 | 96.78 | 92.05 | 47.42 |
| HO1 | 7.4 |  | 43147612 | 41526926 | 6.23G | 0.02 | 96.95 | 92.42 | 46.62 |
| HO2 | 6.6 |  | 42250478 | 40616298 | 6.09G | 0.02 | 96.87 | 92.29 | 46.32 |
| HO3 | 6.6 |  | 50304726 | 48365314 | 7.25G | 0.02 | 96.91 | 92.37 | 45.82 |

| Database | Number of Unigenes | Percentage (%) |
| --- | --- | --- |
| NR | 114104 | 60.64 |
| NT | 48271 | 25.65 |
| KO | 51137 | 27.17 |
| SwissProt | 95837 | 50.93 |
| PFAM | 98396 | 52.29 |
| GO | 100502 | 53.41 |
| KOG | 46701 | 24.82 |
| All^1^ | 17500 | 9.30 |
| at least one^2^ | 135102 | 71.80 |

**Table S4. Summary of annotations**

^1^ Annotated in all databases

^2^ Annotated in at least one database

**Table S5. Comparisons between sample reads and Trinity assembly**

| Sample name | Total reads | Total mapped |
| --- | --- | --- |
| LY1 | 43147222 | 33771780(78.27%) |
| LY2 | 45578352 | 34796980(76.35%) |
| LY3 | 41883522 | 32557622(77.73%) |
| HY1 | 48004270 | 34272470(71.39%) |
| HY2 | 49320128 | 38248492(77.55%) |
| HY3 | 52128974 | 41059506(78.77%) |
| LO1 | 42914578 | 32855368(76.56%) |
| LO2 | 53474142 | 39428476(73.73%) |
| LO3 | 50699804 | 37999224(74.95%) |
| HO1 | 41526926 | 32661242(78.65%) |
| HO2 | 40616298 | 30733510(75.67%) |
| HO3 | 48365314 | 37658118(77.86%) |

**Table S6. DEGs statistics between different groups**

|  | HY vs LY | HO vs LO | LO vs LY | HO vs HY |
| --- | --- | --- | --- | --- |
| up | 547 | 186 | 4915 | 79 |
| down | 717 | 1860 | 1523 | 60 |
| all | 1264 | 2046 | 6438 | 139 |

**Table S7-1. Repeatability of sampling and processing.**

| Extracted Ions | No. | Retention time | Peak area | Mass accuracy |
| --- | --- | --- | --- | --- |
| 255.0510 | 1 | 3.083141191 | 377190.2818 | 255.0511143 |
|  | 2 | 3.045430195 | 436341.0855 | 255.0510352 |
|  | 3 | 3.063714241 | 506944.3963 | 255.0515931 |
|  | 4 | 3.055670803 | 314524.8581 | 255.0516997 |
|  | 5 | 2.964822359 | 407989.8187 | 255.0519056 |
|  | 6 | 3.072758898 | 399113.8388 | 255.0513843 |
|  | RSD (%) | 1.398133932 | 15.67539999 | 0.000133461 |
| 609.1461 | 1 | 8.580634747 | 1507255.652 | 609.1448537 |
|  | 2 | 8.601243528 | 1377446.834 | 609.1431049 |
|  | 3 | 8.567729715 | 1648460.598 | 609.145954 |
|  | 4 | 8.555658161 | 1357962.182 | 609.1452194 |
|  | 5 | 8.584435488 | 1478873.744 | 609.1467241 |
|  | 6 | 8.557274107 | 1440666.66 | 609.1458453 |
|  | RSD (%) | 0.205282957 | 7.16043113 | 0.000204723 |
| 423.1297 | 1 | 11.02675607 | 539247.984 | 423.1290533 |
|  | 2 | 11.04325898 | 485972.248 | 423.127862 |
|  | 3 | 11.03258358 | 597021.8787 | 423.129574 |
|  | 4 | 11.01755565 | 538291.2804 | 423.1291514 |
|  | 5 | 11.02224064 | 496876.4494 | 423.129343 |
|  | 6 | 11.01490157 | 517364.9089 | 423.1292977 |
|  | RSD (%) | 0.095130154 | 7.482138941 | 0.000143493 |
| 531.2728 | 1 | 17.06996808 | 130747.268 | 531.2794363 |
|  | 2 | 17.06735764 | 127050.0824 | 531.2777492 |
|  | 3 | 17.06006976 | 130799.823 | 531.2805065 |
|  | 4 | 17.07639484 | 120964.7853 | 531.2797377 |
|  | 5 | 17.05833677 | 103436.8311 | 531.2807184 |
|  | 6 | 17.06918132 | 108920.8951 | 531.2808105 |
|  | RSD (%) | 0.039301777 | 9.687031266 | 0.000217907 |
| 303.2330 | 1 | 23.60735949 | 539323.7939 | 303.2325991 |
|  | 2 | 23.61682697 | 496094.3603 | 303.232258 |
|  | 3 | 23.60261539 | 620426.8939 | 303.2330556 |
|  | 4 | 23.59938494 | 511851.8905 | 303.2330417 |
|  | 5 | 23.60707945 | 501302.4401 | 303.233445 |
|  | 6 | 23.61586757 | 490709.9643 | 303.2331679 |
|  | RSD (%) | 0.029594445 | 9.318803348 | 0.000140782 |
| 369.2433 | 1 | 25.57036165 | 2618402.003 | 369.242414 |
|  | 2 | 25.57158249 | 2342725.109 | 369.2421737 |
|  | 3 | 25.56516981 | 2775872.174 | 369.2433189 |
|  | 4 | 25.5644477 | 2492447.839 | 369.2428906 |
|  | 5 | 25.5729883 | 2437842.171 | 369.2433992 |
|  | 6 | 25.58614553 | 2448087.073 | 369.2433992 |
|  | RSD (%) | 0.030659219 | 6.130401857 | 0.000144899 |

**Table S7-2. Intra-day variability of the UPLC-QTOF/MS method**

| Extracted Ions | No. | Retention time | Peak area | Mass accuracy |
| --- | --- | --- | --- | --- |
| 255.0510 | 1 | 3.057932311 | 320172.2549 | 255.0514302 |
|  | 2 | 3.051170672 | 314672.5482 | 255.0506198 |
|  | 3 | 3.05243339 | 321206.5205 | 255.0506909 |
|  | 4 | 3.134048812 | 320835.0866 | 255.0511032 |
|  | 5 | 3.067360398 | 314155.2978 | 255.0507475 |
|  | 6 | 2.971436021 | 314930.4232 | 255.0507163 |
|  | RSD (%) | 1.694852464 | 1.068733468 | 0.000124006 |
| 609.1461 | 1 | 8.57635191 | 1372791.946 | 609.1457133 |
|  | 2 | 8.575390391 | 1302929.935 | 609.1437895 |
|  | 3 | 8.587600151 | 1403589.254 | 609.1446882 |
|  | 4 | 8.571939018 | 1365360.524 | 609.1449175 |
|  | 5 | 8.591185511 | 1420715.075 | 609.1442147 |
|  | 6 | 8.582720058 | 1350832.722 | 609.143354 |
|  | RSD (%) | 0.0880353 | 3.027372981 | 0.000138601 |
| 423.1297 | 1 | 11.04634411 | 540436.8163 | 423.1289009 |
|  | 2 | 11.02303398 | 519078.0781 | 423.1274012 |
|  | 3 | 11.0298883 | 523561.4957 | 423.1280692 |
|  | 4 | 11.02143378 | 525823.2214 | 423.1282112 |
|  | 5 | 11.03552297 | 517737.8677 | 423.1277741 |
|  | 6 | 11.02373471 | 510186.1777 | 423.1278198 |
|  | RSD (%) | 0.086846768 | 1.949448595 | 0.000120413 |
| 531.2728 | 1 | 17.05744053 | 116110.964 | 531.2808417 |
|  | 2 | 17.06782716 | 125962.0042 | 531.2784494 |
|  | 3 | 17.05053297 | 122784.4566 | 531.2789687 |
|  | 4 | 17.06025938 | 117763.4897 | 531.2798206 |
|  | 5 | 17.06598731 | 121478.3921 | 531.2789087 |
|  | 6 | 17.06955818 | 126025.4806 | 531.2779829 |
|  | RSD (%) | 0.042448044 | 3.384926904 | 0.000192959 |
| 303.2330 | 1 | 23.62567473 | 497009.9893 | 303.2331908 |
|  | 2 | 23.61784849 | 502031.3715 | 303.2322522 |
|  | 3 | 23.61374951 | 474145.9429 | 303.232354 |
|  | 4 | 23.61824427 | 507950.9549 | 303.2328131 |
|  | 5 | 23.61832605 | 494243.1731 | 303.2324233 |
|  | 6 | 23.61988327 | 486855.7479 | 303.2319001 |
|  | RSD (%) | 0.016423848 | 2.41867859 | 0.00014917 |
| 369.2433 | 1 | 25.5922553 | 2467737.874 | 369.2435915 |
|  | 2 | 25.59703004 | 2420219.721 | 369.2421713 |
|  | 3 | 25.57858614 | 2470742.639 | 369.2418241 |
|  | 4 | 25.59218798 | 2522129.482 | 369.2431381 |
|  | 5 | 25.5890432 | 2448165.443 | 369.2422422 |
|  | 6 | 25.59523399 | 2495865.023 | 369.2409941 |
|  | RSD (%) | 0.025609202 | 1.441532057 | 0.000251673 |

**Table S7-3. Inter-day variability of the UPLC-QTOF/MS method**

| Extracted Ions | No. | Retention time | Peak area | Mass accuracy |
| --- | --- | --- | --- | --- |
| 255.0510 | 1 | 3.134048812 | 320835.0866 | 255.0511032 |
|  | 2 | 3.067360398 | 314155.2978 | 255.0507475 |
|  | 3 | 2.971436021 | 314930.4232 | 255.0507163 |
|  | 4 | 3.076107123 | 299367.8167 | 255.0511315 |
|  | 5 | 3.119415882 | 299005.7919 | 255.0506631 |
|  | 6 | 3.076891573 | 298206.2994 | 255.0504806 |
|  | 7 | 3.007019733 | 242243.027 | 255.0508862 |
|  | 8 | 3.101155847 | 279325.8247 | 255.0510343 |
|  | 9 | 3.059693377 | 261944.8126 | 255.0510038 |
|  | RSD (%) | 1.684259155 | 8.996487643 | 8.7735E-05 |
| 609.1461 | 1 | 8.571939018 | 1365360.524 | 609.1449175 |
|  | 2 | 8.591185511 | 1420715.075 | 609.1442147 |
|  | 3 | 8.582720058 | 1350832.722 | 609.143354 |
|  | 4 | 8.562201551 | 1030444.465 | 609.1436265 |
|  | 5 | 8.593797336 | 1145102.635 | 609.1440872 |
|  | 6 | 8.602507046 | 987697.676 | 609.1427742 |
|  | 7 | 8.578687717 | 1133199.628 | 609.144954 |
|  | 8 | 8.559843029 | 1373307.564 | 609.1450091 |
|  | 9 | 8.583333618 | 1020797.787 | 609.1452354 |
|  | RSD (%) | 0.166150224 | 14.46216244 | 0.000140803 |
| 423.1297 | 1 | 11.02143378 | 525823.2214 | 423.1282112 |
|  | 2 | 11.03552297 | 517737.8677 | 423.1277741 |
|  | 3 | 11.02373471 | 510186.1777 | 423.1278198 |
|  | 4 | 11.02311816 | 356467.0624 | 423.127133 |
|  | 5 | 11.06131845 | 445469.6423 | 423.1276229 |
|  | 6 | 11.07509739 | 390483.7993 | 423.1273398 |
|  | 7 | 11.05139395 | 431300.4894 | 423.1290053 |
|  | 8 | 11.02714198 | 465236.8249 | 423.1285652 |
|  | 9 | 11.03581559 | 452957.0683 | 423.1287366 |
|  | RSD (%) | 0.172765134 | 12.67823417 | 0.000152383 |
| 531.2728 | 1 | 17.06025938 | 117763.4897 | 531.2795739 |
|  | 2 | 17.06598731 | 121478.3921 | 531.2785776 |
|  | 3 | 17.06955818 | 126025.4806 | 531.2779127 |
|  | 4 | 17.08094617 | 94968.53884 | 531.2782586 |
|  | 5 | 17.09811279 | 94905.30902 | 531.2791891 |
|  | 6 | 17.10023919 | 100689.6383 | 531.2779408 |
|  | 7 | 17.08036164 | 81436.32793 | 531.2791383 |
|  | 8 | 17.0679066 | 110463.5264 | 531.280151 |
|  | 9 | 17.07415309 | 77525.82996 | 531.2804074 |
|  | RSD (%) | 0.081720294 | 16.82749249 | 0.000172505 |
| 303.2330 | 1 | 23.61824427 | 507950.9549 | 303.2328131 |
|  | 2 | 23.61832605 | 494243.1731 | 303.2324233 |
|  | 3 | 23.61988327 | 486855.7479 | 303.2319001 |
|  | 4 | 23.59827 | 311393.1974 | 303.2322713 |
|  | 5 | 23.59480619 | 383107.8815 | 303.2322544 |
|  | 6 | 23.60829216 | 335724.9562 | 303.2319288 |
|  | 7 | 23.6063927 | 413224.657 | 303.2328022 |
|  | 8 | 23.6106292 | 467023.6328 | 303.2326174 |
|  | 9 | 23.61624204 | 452701.9971 | 303.2328507 |
|  | RSD (%) | 0.038428942 | 16.66880465 | 0.00012148 |
| 369.2433 | 1 | 25.59218798 | 2522129.482 | 369.2427634 |
|  | 2 | 25.5890432 | 2448165.443 | 369.2421928 |
|  | 3 | 25.59523399 | 2495865.023 | 369.2416404 |
|  | 4 | 25.48038128 | 2872437.614 | 369.2427087 |
|  | 5 | 25.49403885 | 2992909.045 | 369.2424061 |
|  | 6 | 25.53141345 | 2864965.612 | 369.2415041 |
|  | 7 | 25.56559337 | 2702650.091 | 369.243186 |
|  | 8 | 25.58260299 | 2545609.911 | 369.2428487 |
|  | 9 | 25.59612285 | 2555144.52 | 369.24277 |
|  | RSD (%) | 0.177448796 | 7.432846095 | 0.000153992 |

**Table S8. LC-MS identification results of other polyphenols in the phenylpropanoid biosynthesis.**

| **Compound Name** | **CAS** | **Formula** | **Mass** | **Adduct Type** | **Theoretical m/z** | **Retention time (min)** | **Fragment information** |
| --- | --- | --- | --- | --- | --- | --- | --- |
| **Sinapic acid** | **530-59-6/7362-37-0** | **C11H12O5** | **224.2100** | **M-H** | **223.0612** | **6.66** | **121.03,149.02,193.01,163.03,223.06** |
| **Sinapyl alcohol** | **537-33-7** | **C11H14O4** | **210.2265** | **M-H** | **209.0819** | **6.67** | **179.03,121.03,151.03,161.02** |
| **p-Coumaric acid** | **7400-08-0/501-98-4** | **C9H8O3** | **164.1580** | **M-H** | **163.0401** | **6.75** | **119.05,117.04,145.90,104.03,162.84** |
| **beta-D-Glucosyl-2-hydroxycinnamate** | **618-67-7/2446-60-8** | **C15H18O8** | **326.2986** | **M-H** | **325.0929** | **6.76** | **119.05,163.04** |
| **Coniferyl aldehyde** | **458-36-6** | **C10H10O3** | **178.1846** | **M-H** | **177.0557** | **6.84** | **133.03,105.04,121.03,177.02** |
| **Caffeic acid** | **501-16-6/331-39-5** | **C9H8O4** | **180.1574** | **M-H** | **179.0350** | **6.88** | **135.05,134.04,107.05,117.04** |
| **Ferulic acid** | **537-98-4** | **C10H10O4** | **194.1840** | **M-H** | **193.0506** | **7.80** | **134.04,133.03,106.05,158.85,178.03** |
| **trans-2-Hydroxycinnamate** | **614-60-8** | **C9H8O3** | **164.1580** | **M-H** | **163.0401** | **8.18** | **119.05,117.04,145.89,104.03,162.84** |
| **cis-2-Hydroxycinnamate (Coumarinate)** | **583-17-5** | **C9H8O3** | **164.1580** | **M-H** | **163.0401** | **8.69** | **119.05,117.04,145.89,162.89,104.03** |
| **p-Coumaraldehyde** | **2538-87-6** | **C9H8O2** | **148.1586** | **M-H** | **147.0452** | **15.34** | **119.06,117.03,129.04,147.05** |
| **Secoisolariciresinol** | **29388-59-8** | **C20H26O6** | **362.1729** | **M-H** | **361.1655** | **10.12** | **361.17,165.05,346.14,122.04,179.07** |

The ingredients with two CAS numbers indicated its trans- and/or cis- conformations.
